# Transcriptomic Profiling in Mice With CB1 receptor Deletion in Primary Sensory Neurons Suggests New Analgesic Targets for Neuropathic Pain

**DOI:** 10.3389/fphar.2021.781237

**Published:** 2022-01-03

**Authors:** Yongmin Liu, Min Jia, Caihua Wu, Hong Zhang, Chao Chen, Wenqiang Ge, Kexing Wan, Yuye Lan, Shiya Liu, Yuanheng Li, Mengyue Fang, Jiexi He, Hui-Lin Pan, Jun-Qiang Si, Man Li

**Affiliations:** ^1^ Department of Neurobiology, School of Basic Medicine, Tongji Medical College, Huazhong University of Science and Technology, Wuhan, China; ^2^ Department of Pathophysiology, Medical College of Shihezi University, Shihezi, China; ^3^ Clinical Laboratories, Wuhan First Hospital, Wuhan, China; ^4^ Department of Acupuncture, Wuhan First Hospital, Wuhan, China; ^5^ Department of Anesthesiology and Perioperative Medicine, The University of Texas MD Anderson Cancer Center, Houston, TX, United States; ^6^ Department of Physiology, Medical College of Shihezi University, Shihezi, China

**Keywords:** cannabinoid receptor, transcriptome sequencing, neuropathic pain, dorsal root ganglia, downstream gene, neuroinflammation

## Abstract

Type 1 and type 2 cannabinoid receptors (CB1 and CB2, respectively) mediate cannabinoid-induced analgesia. Loss of endogenous CB1 is associated with hyperalgesia. However, the downstream targets affected by ablation of CB1 in primary sensory neurons remain unknown. In the present study, we hypothesized that conditional knockout of CB1 in primary sensory neurons (CB1cKO) alters downstream gene expression in the dorsal root ganglion (DRG) and that targeting these pathways alleviates neuropathic pain. We found that CB1cKO in primary sensory neurons induced by tamoxifen in adult Advillin-Cre:CB1-floxed mice showed persistent hyperalgesia. Transcriptome/RNA sequencing analysis of the DRG indicated that differentially expressed genes were enriched in energy regulation and complement and coagulation cascades at the early phase of CB1cKO, whereas pain regulation and nerve conduction pathways were affected at the late phase of CB1cKO. Chronic constriction injury in mice induced neuropathic pain and changed transcriptome expression in the DRG of CB1cKO mice, and differentially expressed genes were mainly associated with inflammatory and immune-related pathways. Nerve injury caused a much larger increase in CB2 expression in the DRG in CB1cKO than in wildtype mice. Interfering with downstream target genes of CB1, such as antagonizing CB2, inhibited activation of astrocytes, reduced neuroinflammation, and alleviated neuropathic pain. Our results demonstrate that CB1 in primary sensory neurons functions as an endogenous analgesic mediator. CB2 expression is regulated by CB1 and may be targeted for the treatment of neuropathic pain.

## Introduction

Neuropathic pain, caused by a lesion or disease affecting the somatosensory system, is a major clinical problem and has a considerable impact on the life quality of patients (Burke et al., 2017). The antinociceptive efficacy of cannabinoids has been unequivocally demonstrated in several models of neuropathic pain in animal studies (Walker and Hohmann, 2005). However, clinical application of cannabinoids is severely hindered by adverse reactions resulting from its central actions, such as cognitive deficits, memory impairment, motor disturbances, addiction, and cognitive impairment (Hossain et al., 2020).

Analgesic properties of cannabinoids are achieved mainly by activating G protein-coupled receptors, i.e., type 2 cannabinoid receptor (CB2) and type 1 cannabinoid receptor (CB1) (Matsuda et al., 1990; Munro et al., 1993). CB1 is mainly at the presynapse, where the activation of CB1 can suppress presynaptic neurotransmitter release through a short-term decrease in Ca2+ influx (Katona and Freund, 2012; Lutz et al., 2015; Lutz, 2020). Nerve injury induces long-lasting CB1 downregulation in dorsal root ganglion (DRG) neurons and diminishes the analgesic effect of the CB1 agonist on neuropathic pain (Luo et al., 2020), which is thought to be attributed to the reversed inhibition by endogenous cannabinoids. CB2 expressed on immune cells and the nervous system has also been implicated in cannabinoid analgesia (Walker and Hohmann, 2005; Ibrahim et al., 2006). CB2 was strongly upregulated in response to various insults, stroke, neuroinflammation, and chronic pain (e.g., Leung, 2004; Solas et al., 2013). Some previous studies reported that CB2 was mainly involved in analgesia through limiting neuroinflammation (Nackley et al., 2003; Elmes et al., 2005; Shang and Tang, 2017). Studies of global-knockout mice have confirmed that CB1 and CB2 were involved in cannabinoid-mediated analgesia (Ibrahim et al., 2006; Sain et al., 2009; Sideris et al., 2016). Moreover, after the selective deletion of CB1 in nociceptive (Nav1.8-expressing) sensory neurons, physiological and basal pain sensitivity was exaggerated, showing the significantly reduced reaction latencies to noxious heat and response thresholds to mechanical stimuli (Agarwal et al., 2007).

Peripheral nerve injury is accompanied by alterations in transcription reprogramming in the peripheral nervous system, which subsequently causes altered behaviors in animals. The variation of DRG gene expression is related to pain phenotypes (Bosma et al., 2020; Sun et al., 2020), which can be screened by RNA sequencing for identification of differentially expressed genes (DEGs) and their functional pathways related to neuropathic pain development. Moreover, single-cell sequencing technology revealed the gene expression patterns in subtypes of DRG neurons after peripheral nerve injury (Wang et al., 2021). Although CB1 knockout may exaggerate pain, it remains unknown how transcriptomic profiling changes in mice with CB1 deletion in peripheral sensory neurons and whether the downstream genes of CB1 may provide new analgesic targets in neuropathic pain.

Therefore, in this study, we investigated whether CB1 conditional knockout from peripheral sensory neurons (CB1cKO) in mice may present mechanical allodynia, thermal hyperalgesia, and a change in transcriptome expression in DRG. Then, we determined the effect of chronic constriction injury (CCI) model of CB1cKO mice on pain behavior and transcriptome expression of DRG. Moreover, we screened and analyzed the DEGs and their functional pathways after CCI and revealed the downstream of CB1. Finally, we observed whether intervention of the downstream target of CB1 may alleviate neuropathic pain. Our findings provided new evidence of the role of CB1 in the development of neuropathic pain and the downstream genes of CB1 may serve as therapeutic targets for neuropathic pain.

## Materials and Methods

### Animals

Ethical guidelines of the International Association for the Study of Pain were strictly followed, and the protocol was approved by the local ACUC. All animals were housed at 22°C–24°C based on a 12-h light/dark cycle and were allowed free access to food and water.

C57BL/6J mice were used in the experiments. Adult male C57BL/6J mice (20–25 g; 8-week-old) were provided by Beijing Vital River Laboratory Animal Technology Co. Ltd.

AvCreERT2 (Advillin-CreERT2) mice (Jax Stock No. 026516) expressed a tamoxifen (TMX)-inducible iCre recombinase directed by mouse Avil (advillin) promoter elements. iCre recombinase activity was found in DRG neurons when induced by TMX.

Cnr^1tm1.2Ltz/^J mice (Jax Stock No.036107) and CB1f/f mice have loxP sites flanking the entire coding region of the Cnr1 gene. After crossing Advillin-CreerT2 (AvCreerT2) mice and CNR1TM1.2LTZ/J mice for two generations, tissue/cell-specific knockout homozygous mice were obtained in the F3 generation after TMX induction (treatment details are shown in *Drug Treatment*), which are referred to as CB1cKO mice.

### Identification of Transgenic Mice

Seven CB1cKO mice and seven wild-type littermates male mice (WT mice) were used for genotyping. Before TMX induction, the genomic DNA was extracted from the toes or tails of the genetic mice using chloroform/phenol and precipitated by isopropanol. It was then washed by ethanol (75%) and dissolved in deionized water. The primers for PCR are shown in Supplementary Table S1. The products of PCR reactions are shown in Supplementary Table S2. The products were dissolved on agarose gel (1.5%), stained with ethidium bromide, and photographed.

CB1 in DRG was verified by immunofluorescence and real-time polymerase chain reaction (RT-qPCR) on the 14th day after the final TMX induction (detailed method is shown in *Real-Time Polymerase Chain Reaction* and *Immunofluorescence*).

### Drug Treatment

TMX (Sigma T5648) was dissolved into corn oil and was injected intraperitoneally (i.p.) once every other day, a total of five times, 2 mg/day (10 mg total dose) (Feil et al., 2009; Anastassiadis et al., 2010). Subsequent verification and CCI modeling were performed 14 days after the final injection.

AM1241 (Sigma A6478) is a CB2 agonist, and AM630 (Sigma SML0327) is a highly specific CB2 antagonist. The drug was dissolved in dimethyl sulfoxide (DMSO), Tween 80, and normal saline (the ratio is 1:2:7). To determine the relationship between neuropathic pain and CB2, 36 WT mice were randomly divided into WT CCI + vehicle (DMSO:Tween 80:normal saline = 1:2:7), WT CCI + 0.1, 1, 3 mg/kg AM1241 (Sigma A6478; Wu et al., 2021; Lozano-Ondoua et al., 2010; Ma et al., 2021) groups, and WT CCI + 1, 3 mg/kg AM630 (Sigma SML0327; Malan et al., 2001; Buffon et al., 2020; Sultana et al., 2021) groups (*n* = 6 at each group). Fifteen CB1cKO mice were randomly divided into CB1cKO CCI + vehicle and CB1cKO CCI + 3, 5 mg/kg AM630 (*n* = 5 at each group). AM1241/AM630 or its vehicle (0.1 ml) was injected i.p. daily from 12th to 17th day after CCI surgery.

### Behavioral Testing

Ten CB1cKO mice and 10 WT mice were used to test tactile withdrawal threshold and thermal latency by von Frey and Hargreaves plantar tests before and every 3 days after TMX induction. To rule out the effect of TMX on the nociceptive threshold, WT mice were also i.p. injected with TMX at the same time and dose as CB1cKO mice.

The “up and down” method was used to determine the threshold of tactile withdrawal in mice (Chaplan et al., 1994). After adapting for half an hour, the plantar surface of the left hind paw was stimulated vertically by von Frey filaments (Stoelting, Wood Dale, IL, USA), and the stiffness was increased logarithmically. Positive response was defined as paw flinching or brisk withdrawal after stimulation for 5 s. Thresholds of tactile withdrawal were detected twice, and the average was taken. A hot plate with surface temperature controlled at 53°C was used to determine thermal latency. The withdrawal latency was defined as the interval between the moment mice were placed on the plate and the time point of flick of the paw or a quick withdrawal. In order to prevent tissue damage, 20 s were set as the cutoff time (Hargreaves et al., 1988). The thermal test was repeated three times, with an interval of 10 min, and the average was taken.

### Chronic Constriction Injury Model Establishment

Fourteen WT mice were randomly divided into WT sham and WT CCI (*n* = 7 at each group), and 14 CB1cKO mice (eight male mice and six female mice) were randomly divided into CB1cKO sham and CB1cKO CCI (*n* = 7 at each group). All mice have received TMX induction. After the 14th day of the final TMX injection, the CCI model of neuropathic pain was chosen based on a previous description (Bennett and Xie, 1988). Then, 0.5% pentobarbital sodium was injected i.p. at a dose of 0.2 ml/10 g body weight. Corneal reflex, muscular tension, and respiratory and pain indicators were examined after intraperitoneal injection to ensure the anesthetic effect and the safety of animals. Experiments were conducted after the injection, and the body temperature was maintained by a heating pad during anesthesia. The left sciatic nerve was exposed at the midthigh level. Proximal to the sciatic trifurcation, two ligature knots (4–0 chromic gut) were loosely tied with a spacing of approximately 1 mm. However, in the sham-CCI group, muscles were separated and the sciatic nerve was exposed but not ligated with the chromic gut. The mechanical withdrawal threshold (MWT) and thermal withdrawal latency (TWL) of every mouse were measured as an assessment of nociception on days 0, 3, 5, 7, 10, and 13 after the CCI operation.

### Real-Time Polymerase Chain Reaction

L4–L6 DRGs were harvested and immediately frozen on dry ice and stored at −80°C. TRIzol reagent (Invitrogen) was used to separate the total RNA that was extracted from DRG tissues (rapidly frozen). RT-qPCR was performed to determine the level of target gene and reference gene (gapdh), with Vazyme SYBR Premix Ex Taq II (Perfect Real Time). The total volume of the reaction system was 10 μl, which was composed of SYBR Premix Ex Taq TM(2×) (5 μl), cDNA (1 μl), ddH_2_O (3.6 μl), and specific primer (0.2 μl; 10 μM). Reaction conditions: 95°C for 3 min; 95°C for 8 s, 60°C for 20 s, 40 cycles; melting. Data were normalized based on GAPDH, and the 2^−∆∆^Ct method was used. The primers for PCR are shown in Supplementary Table S3.

### mRNA Library Constructs and Sequencing

Twelve CB1cKO mice and their six WT mice were randomly divided into six groups, including WT14, WT28, CB1cKO14, CB1cKO28, CB1cKO sham28, and CB1cKO CCI28 (*n* = 3 in each group). All mice have received TMX induction. On the 14th day and 28th day after the final injection of TMX, L4–L6 DRG of CB1cKO mice and WT mice was extracted for RNA sequencing, respectively. On the 13th day after CCI, L4–L6 DRG of CB1cKO mice was extracted for RNA sequencing.

mRNA was purified by magnetic beads with oligo (dT) and then fragmented into small pieces at a proper temperature using fragment buffer. The first-strand cDNA was produced by random reverse transcription (hexamer-primed), and the second-strand cDNA was then synthesized. RNA Index Adapters and A-Tailing Mix were added and incubated to terminate repair. PCR was performed for amplification of cDNA fragments harvested in the previous step, and the amplified products were further purified using Ampure XP Beads. The products were dissolved in EB solution and validated on the Agilent Technologies 2100 bioanalyzer for quality control. The double-stranded PCR products harvested were subjected to heating, denaturing, and circularization by the splint oligo sequence, and the library was constructed, with single-strand circle DNA (ssCir DNA) formatted. Amplification of the final library was performed using phi29 for a DNB (DNA nanoball), which had over 300 copies of one molecule. The DNB was loaded into the nanoarray, and single end reads with 50 bases were produced on the BGIseq500 platform (provided by BGI-Shenzhen, China).

### Data Management and Gene Ontology and Kyoto Encyclopedia of Genes and Genomes Pathway Analysis

SOAPnuke (v1.5.2) was used to filter the sequencing data (Li et al., 2008): 1) reads that contained sequencing adapters were removed; 2) reads with a quality base ratio (base quality ≤5) over 20% were removed; and 3) reads with an unknown base (“N” base) ratio over 5% were removed, and the harvested clean reads were stored in FASTQ format. Using HISAT2 (v2.0.4), the reads were mapped to the reference genome (Kim et al., 2015). Then, rMATS (V3.2.5) (Shen et al., 2014) and Ericscript (v0.5.5) (Benelli et al., 2012) were utilized to determine differential splicing genes (DSGs) and fusion genes, respectively. Using Bowtie2 (v2.2.5) (Langmead and Salzberg, 2012), the reads were aligned to the gene set, a database constructed by BGI (Beijing Genomic Institute in Shenzhen), which covered novel, known, noncoding, and coding transcripts. The RSEM (v1.2.12) (Li and Dewey, 2011) was used to calculate the gene expression level. The pheatmap (v1.0.8) was utilized to draw the heatmap based on gene expressions in a variety of samples. The DESeq2 (v1.4.5) (Love et al., 2014) was used for differential expression analysis, with Q value ≤0.05.

Kyoto Encyclopedia of Genes and Genomes (KEGG) pathway and Gene Ontology (GO) analyses were performed to explore the roles of all DE mRNAs. Briefly, GO analysis was carried out to elucidate genetic regulatory networks of interest by forming hierarchical categories based on the biological process (BP), cellular component (CC), and molecular function (MF) of DEGs. In order to determine the phenotypes, KEGG (https://www.kegg.jp/) enrichment and GO (http://www.geneontology.org/) analyses of annotated DEGs were carried out using Phyper (https://en.wikipedia.org/wiki/Hypergeometric distribution) and Hypergeometric test. The significant levels of pathways and terms were corrected by Bonferroni based on Q value ≤0.05.

### Immunofluorescence

Anesthesia of the mice was performed using chloral hydrate (4%; 10 ml/kg, i.p.). The DRGs were fixed in paraformaldehyde (PFA; 4%) and phosphate buffered saline (PBS; 0.1 M, pH 7.4) for 4–8 h, followed by dehydration in PBS (0.1 M) with 20% and 30% sucrose. After embedding in OCT (provided by Miles Inc., Elkhart, IN, USA), the fixed DRG was vertically sectioned (15 μM in thickness). The sections were harvested and mounted on a slide coated by chrome-alum-gelatin. After PBS (0.1 M) rinsing, the section was blocked by triton X-100 (1.2%) and donkey serum (5%) for 2 h at room temperature. Then, the sections were incubated with primary antibodies of rabbit anti-CB1 (Abcam, #ab137410, 1:200 dilution) and glial fibrillary acidic protein (GFAP; Abcam, #ab4674, 1:5,000 dilution) overnight at 4°C. The primary antibodies were incubated with AffiniPure donkey anti-rabbit IgG (Alexa Fluor 488-conjugated, 1:350 dilution, Jackson) and donkey anti-Chicken IgG (Alexa Fluor 488-conjugated, 1:450 dilution, Jackson) and then observed. Images were acquired using a fluorescence microscope (BX51, Olympus, Japan), and NIH ImageJ software (provided by NIH, Bethesda, MD, USA) was used for their analysis.

### Statistical Analysis

The data were expressed as mean ± SEM. Multiple groups were compared by two-way ANOVA with Bonferroni’s *post-hoc* test, and two groups were compared by Student’s *t*-test. p < 0.05 indicated a significant difference.

## Results

### Generation and Verification of CB1cKO Mice

**FIGURE 1:**
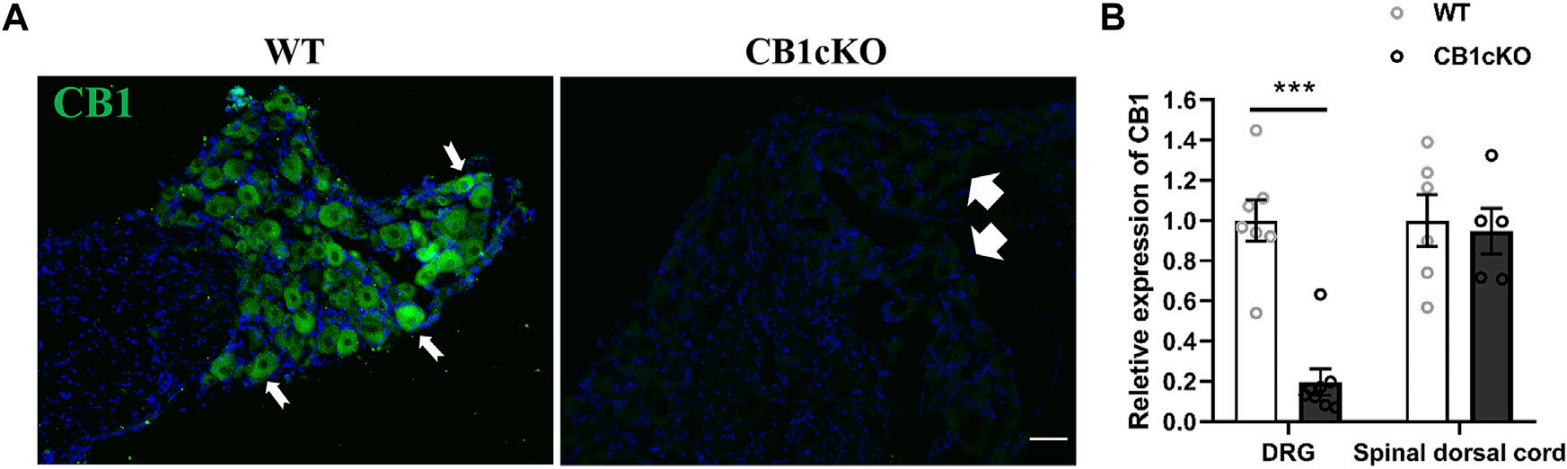
Demonstration of conditional deletion of CB1 in peripheral sensory neuron-specific knockout CB1 mice (CB1cKO). **(A)** Immunofluorescence results of dorsal root ganglion (DRG) sections display the CB1-positive neuron (green) in CB1cKO and wild-type (WT) mice. **(B)** The mRNA relative expression of CB1 in the DRG and dorsal spinal cord on 14 days after the final injection of tamoxifen (TMX). Data are expressed as means ± SEM (*n* = 7 mice/group). The endogenous reference gene is Gapdh, and the mean value in the WT group was set to 1. ***p < 0.001 compared with the WT group at the same time point, two-tailed *t* test. Scale bar, 100 μm.

Before TMX induction, agarose gel electrophoresis images of tail DNA of transgenic mice were shown in Supplementary Figure S1. The knockout efficiency of CB1 in DRG was verified by immunofluorescence and RT-qPCR on the 14th day after the final TMX induction. Immunofluorescence labeling showed that CB1 was expressed in DRG neurons of WT mice and was present on the cell membrane and in the cytoplasm (Figure 1A, indicated by the thin white arrow). More than 90% of CB1 were knocked out on DRG neurons in CB1cKOmice (Figure 1A, thick white arrow). qPCR assay showed that compared with WT mice, CB1 mRNA was significantly reduced in the DRG of CB1cKO mice (Figure 1B, p < 0.001). However, the mRNA level of CB1 in the dorsal spinal horn did not differ significantly between the two groups of mice (Figure 1B, p > 0.05). These results indicated that the expression of CB1 was only reduced in DRG neurons of CB1cKO mice.

### CB1cKO Mice Presented Tactile Allodynia

**FIGURE 2:**
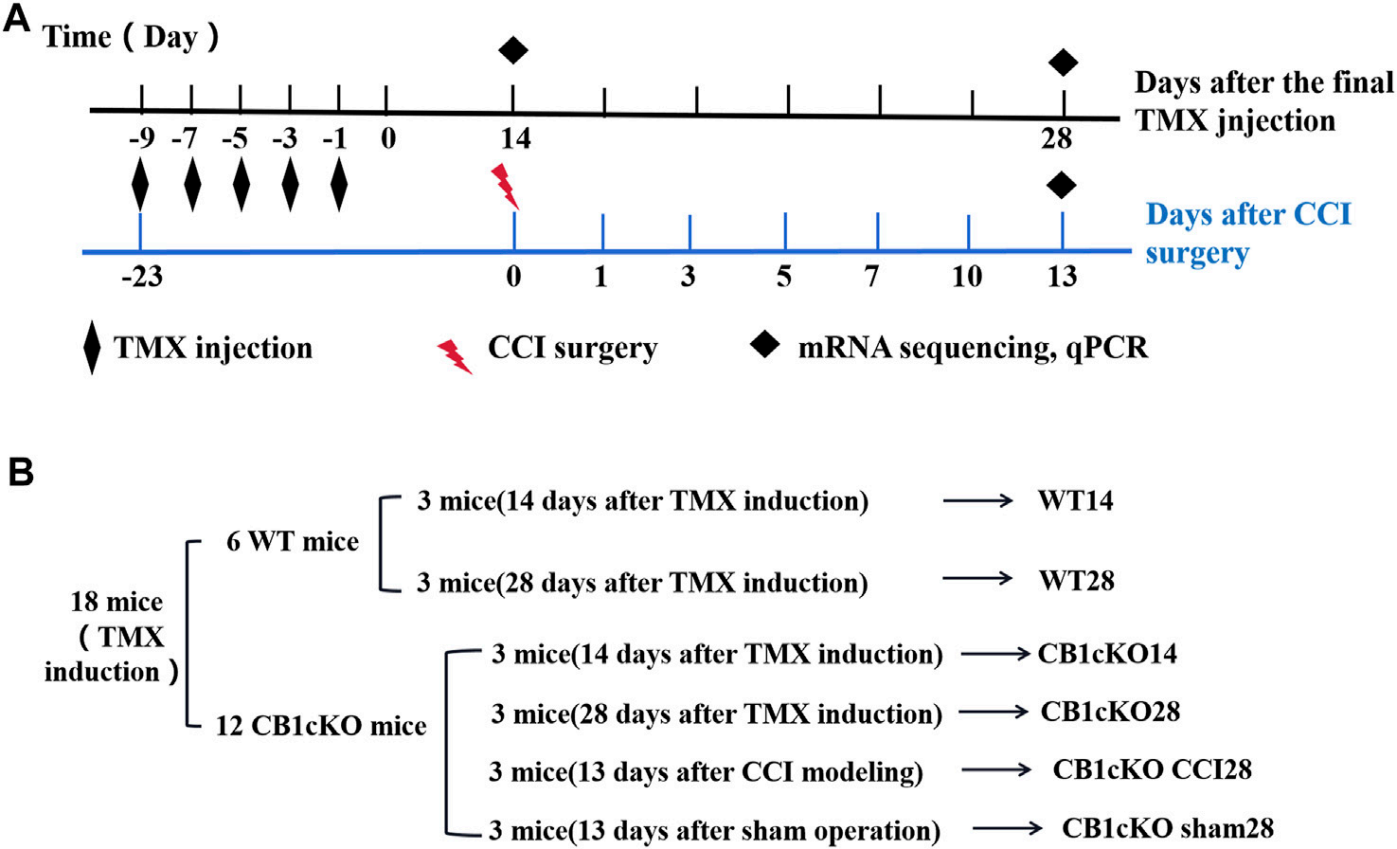
Experimental flowchart and grouping of RNA sequencing (RNA-Seq).

**FIGURE 3:**
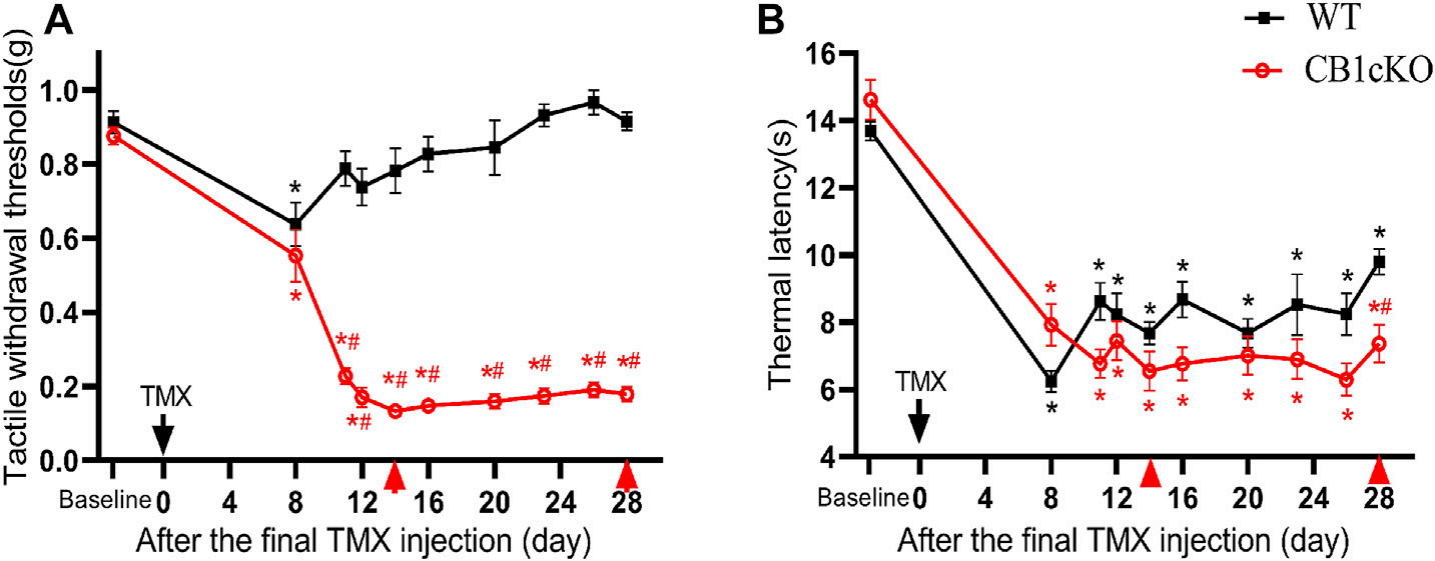
Genetic deletion of CB1 in dorsal root ganglion (DRG) neurons induced tactile allodynia and thermal hyperalgesia. **(A, B)** Time course of the effect of tamoxifen (TMX) induction on the tactile withdrawal thresholds **(A)** and thermal latency **(B)** in CB1cKO and wild-type (WT) mice (*n* = 10 mice/group). Data are expressed as mean ± SEM, *p *<* 0.05, compared with the baseline of the respective group; #p *<* 0.05, compared with the WT group at the same time point (two-way ANOVA with Bonferroni’s *post-hoc* test). The red arrows indicated two time points on day-14 (the early phase of CB1 knockout) and day-28 (the late phase of CB1 knockout) after TMX induction.

Next, we examined nociceptive withdrawal thresholds of CB1cKO mice after TMX induction. An experimental design time line is presented in Figure 2A. CB1cKO mice had a normal mechanical withdrawal threshold (MWT) and thermal latency before TMX induction (Figures 3A, B). After TMX induction, the MWT of CB1cKO mice was significantly reduced from the eighth day to the 28th day after the final TMX injection and reached the lowest level on the 14th day after the final TMX injection (day-14) and remained stable, lasting until day-28 (*p < 0.05, Figure 3A). Compared with WT mice, CB1cKO mice had significantly reduced MWT from day-11 to day-28, showing that physiological, basal pain sensitivity was exaggerated in CB1cKO mice (#p < 0.05, Figure 3A). We also found that the MWT of WT mice has decreased temporarily on day-8 after the same dose of TMX induction and then returned to normal. Notably, TMX induced long-lasting thermal hyperalgesia in both WT mice and CB1cKO mice (*p < 0.05, Figure 3B). So, we only observed a significant difference in the thermal latency between CB1cKO mice and WT mice on day-28 (#p < 0.05, Figure 3B). Together, these results showed that knocking down CB1 in DRG induced tactile allodynia and thermal hyperalgesia. In addition, TMX also caused tactile allodynia and thermal hyperalgesia, but the MWT could return to normal on day-14 in WT mice.

### Differentially Expressed Gene Profile and Functional Enrichment Analysis After CB1 Knockdown

To gain molecular insights into persistent tactile allodynia that are mediated by CB1 of DRG, we conducted mRNA sequencing (mRNA-Seq) analysis. Detailed grouping information and experimental processing are presented in Figures 2A and B. In this project, 18 samples were measured using the DNBSEQ platform, and each sample produced an average of 6.59G data. The average sample ratio for the genome was 87.69% and 74.54% for the gene set. A total of 18,901 genes were detected. The raw data in this study are available in the NCBI SRA database.

**FIGURE 4:**
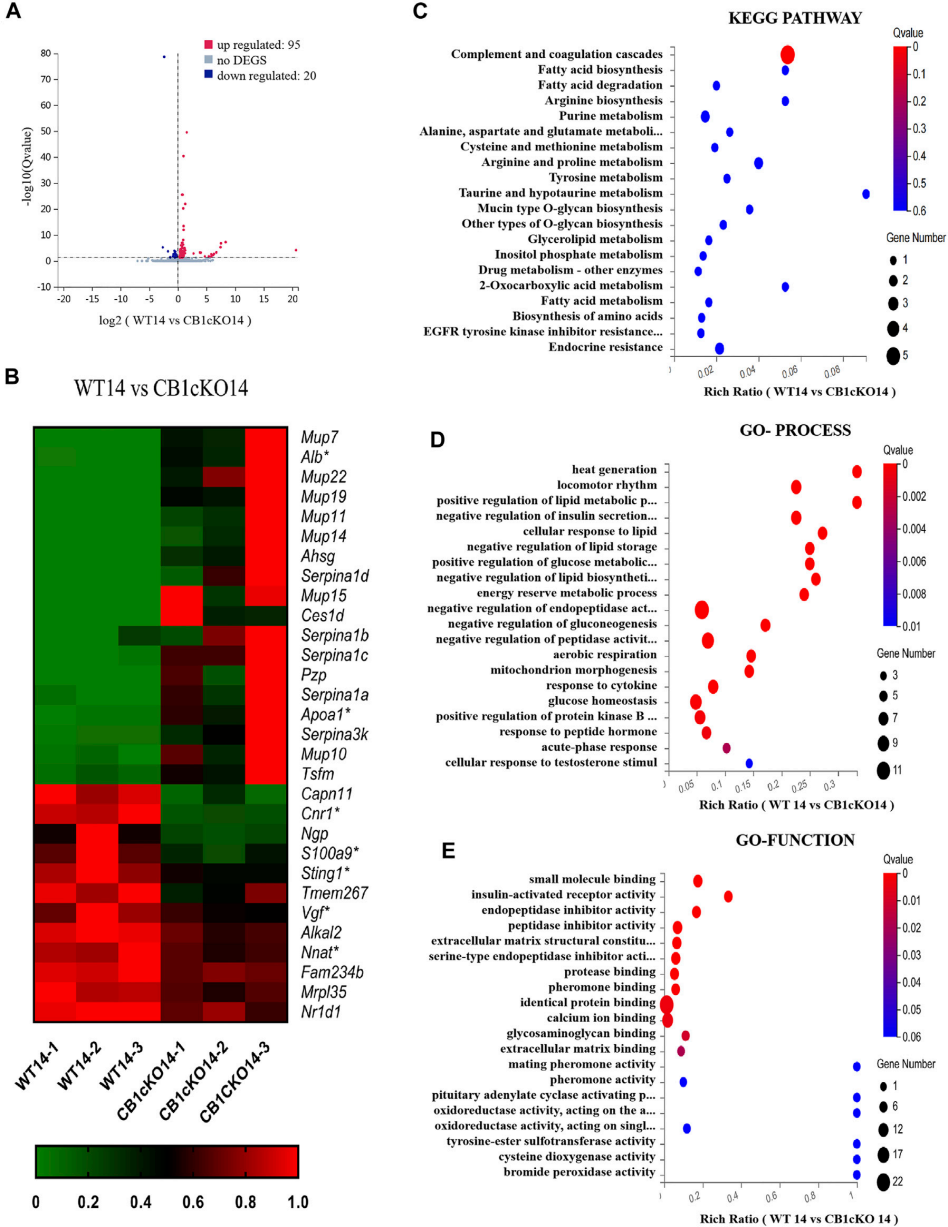
Transcriptomic changes and functional analysis of dorsal root ganglion (DRG) in early CB1 knockdown of peripheral sensory neurons (WT14 vs. CB1cKO14). **(A)** Volcano plot indicated the upregulated and downregulated differentially expressed genes (DEGs) in the early phase of CB1 knockdown. **(B)** Heatmap of the top 20 upregulated and downregulated DEGs in the early phase of CB1 knockout. *This gene is related to pain. #This gene is related to inflammation. **(C–E)** Kyoto Encyclopedia of Genes and Genomes (KEGG) **(C)**, Gene Ontology (GO)-biological processes **(D)**, and GO-molecular function **(E)** enrichment analysis of the DEGs in the early phase of CB1 knockout.

In the early phase of CB1cKO (14 days after the final injection of TMX), compared with WT mice (WT14), 95 mRNAs were upregulated and 20 mRNAs were downregulated in CB1cKO mice (CB1KO 14, Figure 4A); the upregulated DEGs were mainly major urinary protein family (*mup7*, *10*, *11*, *19*, *22*), peptidase inhibitor (*Serpina1a*, *1b*, *1c*, *1d*), and enzymes required for material metabolism. Figure 4B lists the top 20 upregulated and top 10 downregulated DEGs (detailed information on DEGs is shown in Supplementary Table S4). Interestingly, most of them returned to normal after 2 weeks, such as major urinary protein family and peptidase inhibitor.

To better understand the associated functions of the DEGs in DRG after CB1 knockdown, KEGG and GO enrichment analyses of 115 DEGs were performed to identify the most relevant KEGG pathway, biological processes (GO-Process), and molecular functions (GO-Function). Specifically, the DEGs were mostly enriched related to complement and coagulation cascades in the KEGG pathway (p < 0.05, Figure 4C). The biological process of DEGs was mainly involved in heat generation; locomotor rhythm; synthesis and metabolism of glucose, fat, and energy; and negative regulation of endopeptidase or peptidase action (p < 0.01, Figure 4D). The molecular function of DEGs mainly associated with identical protein binding, calcium ion binding, and endopeptidase inhibitor activity (p < 0.01, Figure 4E). These results suggested that imbalances of material metabolism and energy regulation were the stress responses in the early phase of CB1 knockdown.

**FIGURE 5:**
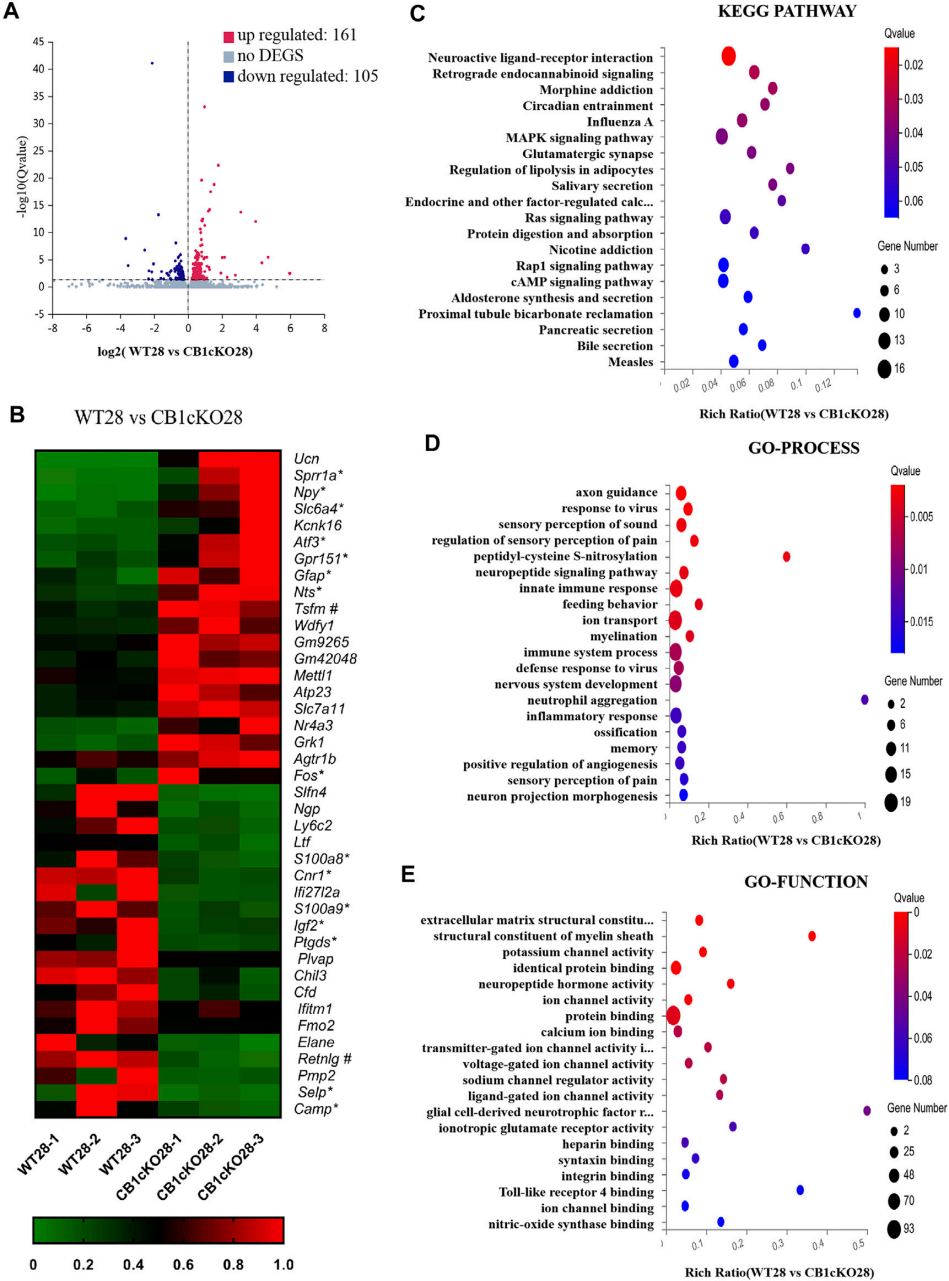
Transcriptomic changes and functional analysis of dorsal root ganglion (DRG) in late CB1 knockdown of peripheral sensory neurons (WT28 vs. CB1cKO28). **(A)** Volcano plot indicated the upregulated and downregulated differentially expressed genes (DEGs) in the late phase of CB1 knockdown. **(B)** Heatmap of the top 20 upregulated and downregulated DEGs in the late phase of CB1 knockout. *This gene is related to pain. #This gene is related to inflammation. **(C–E)** Kyoto Encyclopedia of Genes and Genomes (KEGG) **(C)**, Gene Ontology (GO)-biological processes **(D)**, and GO-molecular function **(E)** enrichment analysis of the DEGs in the late phase of CB1 knockdown.

Next, we screened for 266 DEGs in the late phase of CB1cKO (WT28 vs. CB1cKO28); 161 mRNAs were upregulated, and 105 mRNAs were downregulated (Figure 5A), 209 of which did not change in the early phase of CB1cKO. Many of them were closely related to pain, such as *npy*, *sprr1a*, *gpr151*, *nts*, and so on. Figure 5B lists the top 20 upregulated and downregulated DEGs (detailed information of DEGs is shown in Supplementary Table S5).

Furthermore, we analyzed and predicted the functional pathways of these DEGs of the late phase of CB1cKO. The most significantly enriched KEGG pathways were neuroactive ligand–receptor interaction, retrograde endocannabinoid signaling, morphine addiction, and circadian rhythm (p < 0.05, Figure 5C), and the specific genes in the pathway are listed in Supplementary Table S6. GO-processes analysis suggested that most of the altered genes were involved in axon guidance, regulation of sensory perception of pain, neuropeptide signaling pathway, ion transport, sensory perception of pain, and innate immune response (p < 0.05, Figure 5D), and the specific genes of biological processes are listed in Supplementary Table S8. GO-function analysis suggested that most of the altered genes were associated with protein binding, potassium channel activity, neuropeptide hormone activity, and structural constituent of myelin sheath (p < 0.05, Figure 5E). These results suggest that nerve conduction and sensory perception of pain are mainly a response to the late phase of CB1 knockdown.

### Chronic Constriction Injury Induced More Severe Neuropathic Pain and Alteration of Transcriptomic Profiling in CB1cKO Mice

**FIGURE 6:**
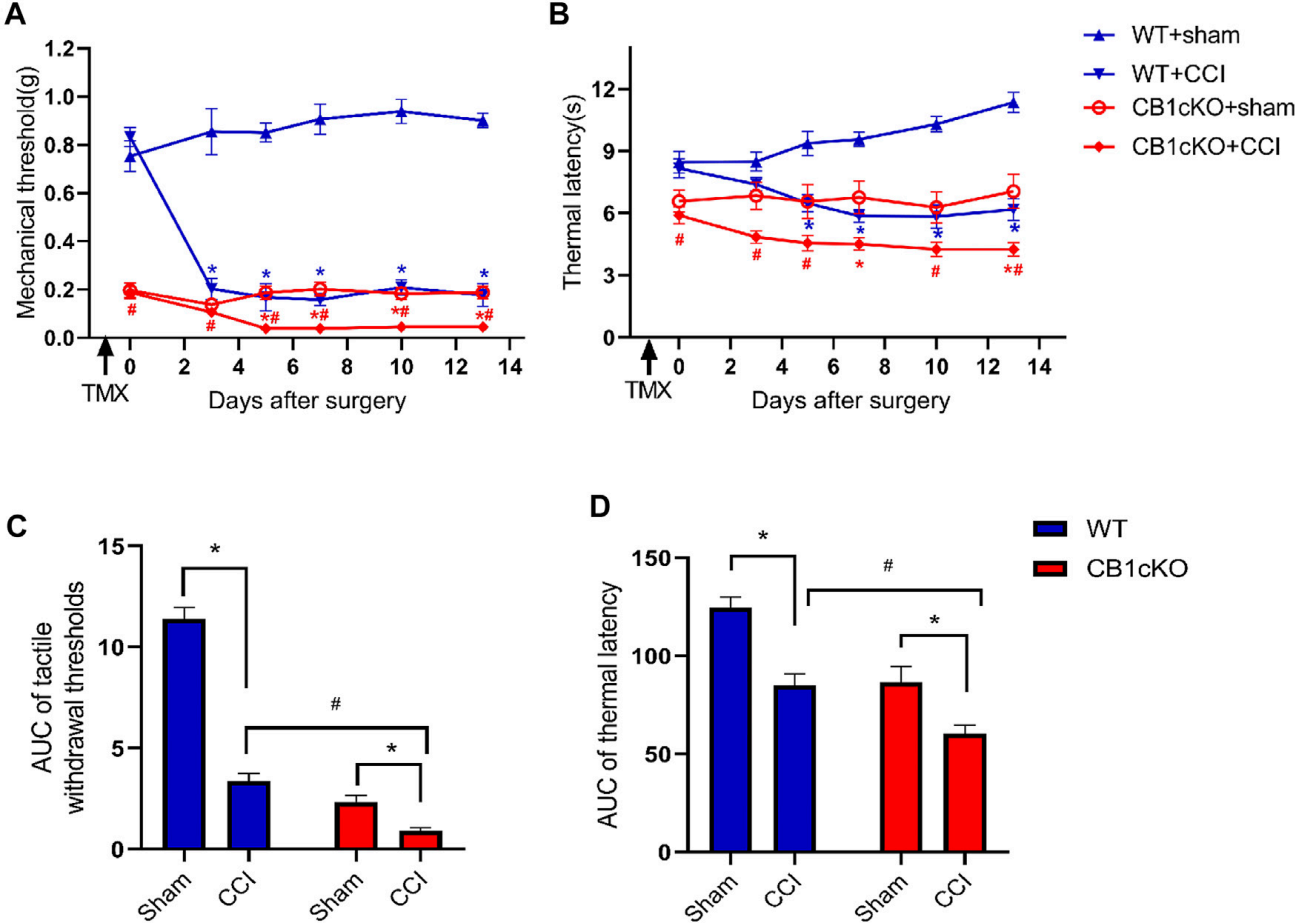
Ablation of CB1 in dorsal root ganglion (DRG) neurons exaggerated tactile allodynia and thermal hyperalgesia induced by nerve injury. **(A, C)** Time course of the tactile withdrawal thresholds in response to von Frey filaments [represented as integrated area under the curve (AUC) in panel **C**] in wild-type (WT) and CB1cKO mice after sham or chronic constriction injury (CCI) surgery. **(B, D)** Time course of the thermal latency to hot plate (represented as integrated AUC in panel **D**) in WT and CB1cKO mice after sham or CCI surgery (*n* = 7 mice/group). Data are represented as mean ± SEM, *p *<* 0.05, compared with the baseline of the respective group; #p < 0.05, compared with WT CCI group at the same time point (two-way ANOVA followed by Bonferroni’s *post-hoc* test).

According to the above results, lack of CB1 in DRG neurons can lead to mechanical allodynia and thermal sensitivity. How does CB1cKO affect the development of neuropathic pain? Therefore, we detected the responses to nociceptive stimuli in both CB1cKO mice and WT mice after CCI. Fourteen CB1cKO mice and their 14 WT littermates were randomly divided into sham and CCI groups, respectively. All mice received CCI surgery or sham treatment on day-14 (14 days after the final TMX injection). The experimental process is shown in Figure 2A. Both CB1cKO and WT mice showed reduced latency to mechanical stimuli and thermal stimuli applied with von Frey and Hargreaves plantar tests in comparison with sham-treated littermates of the same genotype (Figures 6A, B; *p < 0.05). Compared with WT CCI mice, CB1cKO had lower MWT and thermal latency after 5 days of CCI (0.24 g in WT CCI group and 0.04 g in CB1cKO CCI group; Figures 6A, B; #p < 0.05). Moreover, the area under the response-vs.-time curve (AUC) revealed an exaggerated mechanical hypersensitivity and thermal hyperalgesia in CB1cKO mice as compared with WT mice after CCI (Figures 6C, D; #p < 0.05). Thus, our results suggested that peripheral nerve damage could cause more severe neuropathic pain in CB1cKO mice.

**FIGURE 7:**
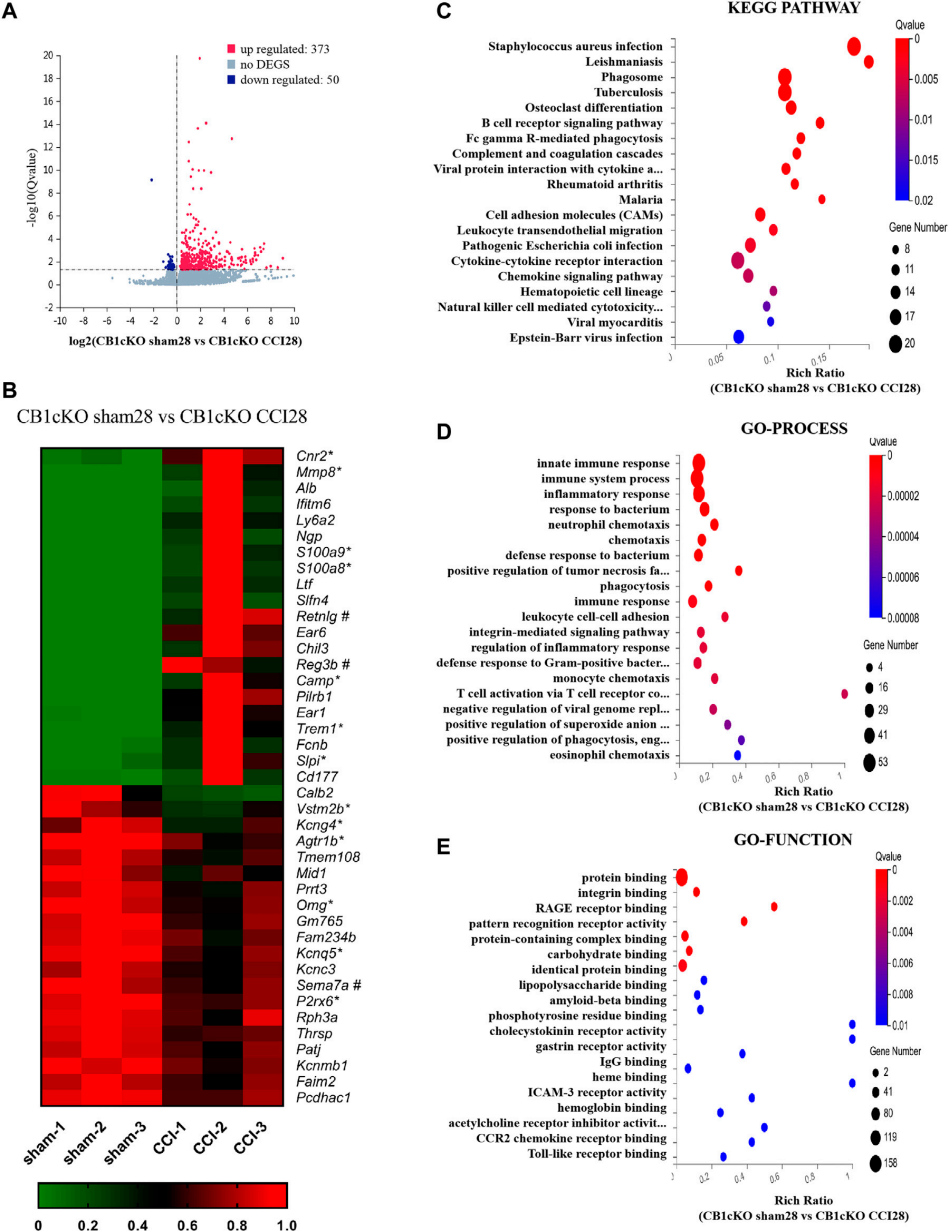
Transcriptomic changes and functional analysis of dorsal root ganglion (DRG) in CB1cKO mice subjected to chronic constriction injury (CCI) (CB1cKO sham28 vs. CB1cKO CCI28). **(A)** Volcano plot indicated the upregulated and downregulated differentially expressed genes (DEGs) in CB1cKO mice after CCI modeling. **(B)** Heatmap of the top 20 upregulated and downregulated DEGs in CB1cKO mice after CCI modeling. *This gene is related to pain. #This gene is related to inflammation. **(C–E)** Kyoto Encyclopedia of Genes and Genomes (KEGG) **(C)**, Gene Ontology (GO)-biological processes **(D)**, and GO-molecular function **(E)** enrichment analysis of the DEGs in CB1cKO mice subjected to CCI.

Fourteen days after CCI modeling, we collected the DRG tissue and sent it for mRNA sequencing, and the results showed that compared with CB1cKO mice of sham operation (CB1KOsham28), 373 genes were upregulated and 50 genes were downregulated in CB1cKO mice of CCI (CB1cKO CCI28) (Figure 7A). The upregulated genes were almost always associated with inflammation and immunity, such as chemokine (C-C motif) and chemokine receptor family, complement component, cytochrome, and interleukin family and receptors, and the difference multiples of the top 20 genes were all more than six times, while some of the downregulated genes were associated with potassium channels. Figure 7B lists the top 20 upregulated and downregulated DEGs (detailed information of DEGs is shown in Supplementary Table S6). Interestingly, we found that compared with CB1cKO mice of sham operation, the expression of DRG CB2 increased significantly by 5.7 times after CCI (Figure 7B, its gene name is *cnr2*). We also found that many glial markers were significantly increased after CCI in CB1cKO mice, such as *cd68*, *gfap*, *csf1*, and *csf1r*. It suggested that the overactivation of CB2 and glial cells may mediate neuropathic pain after CB1 knockout.

Furthermore, we analyzed the functional pathway of 433 DEGs of CB1cKO DRG under neuropathic pain conditions. KEGG analysis revealed that the DEGs were significantly enriched in bacterial or viral infections and immune response, including B-cell receptor signaling pathway, Fc gamma R-mediated phagocytosis, complement and coagulation cascades (Figure 7C, p < 0.001), and the specific genes in the pathway are listed in Supplementary Table S9. Significantly different biological processes of these DEGs also focused on immune response (*csf1*, *csf1r*, *npy*, *cfd*, and *c3*), inflammatory response (*s100a8/s100a9*, *ccr1*, *ccr2*, *pld4*, *adam8*, and *agtr1b*), and defense response to bacteria (*mnda*, *ly6a*, *cfd*, and *fabp4*) (Figure 7D, p < 0.001), and the specific genes of biological processes are listed in Supplementary Table S10. The most significantly enriched molecular functions were concentrated in protein binding, integrin binding, and the binding of various receptors (p < 0.01, Figure 7E). Since GO and KEGG pathway analyses pointed to immune response and inflammatory responses, it suggested that the neuropathic pain of CB1cKO mice with CCI was mediated by a complex and severe neuroinflammatory immune response.

### Targeting Downstream Genes of CB1, Such as CB2, Alleviates Neuropathic Pain

**FIGURE 8:**
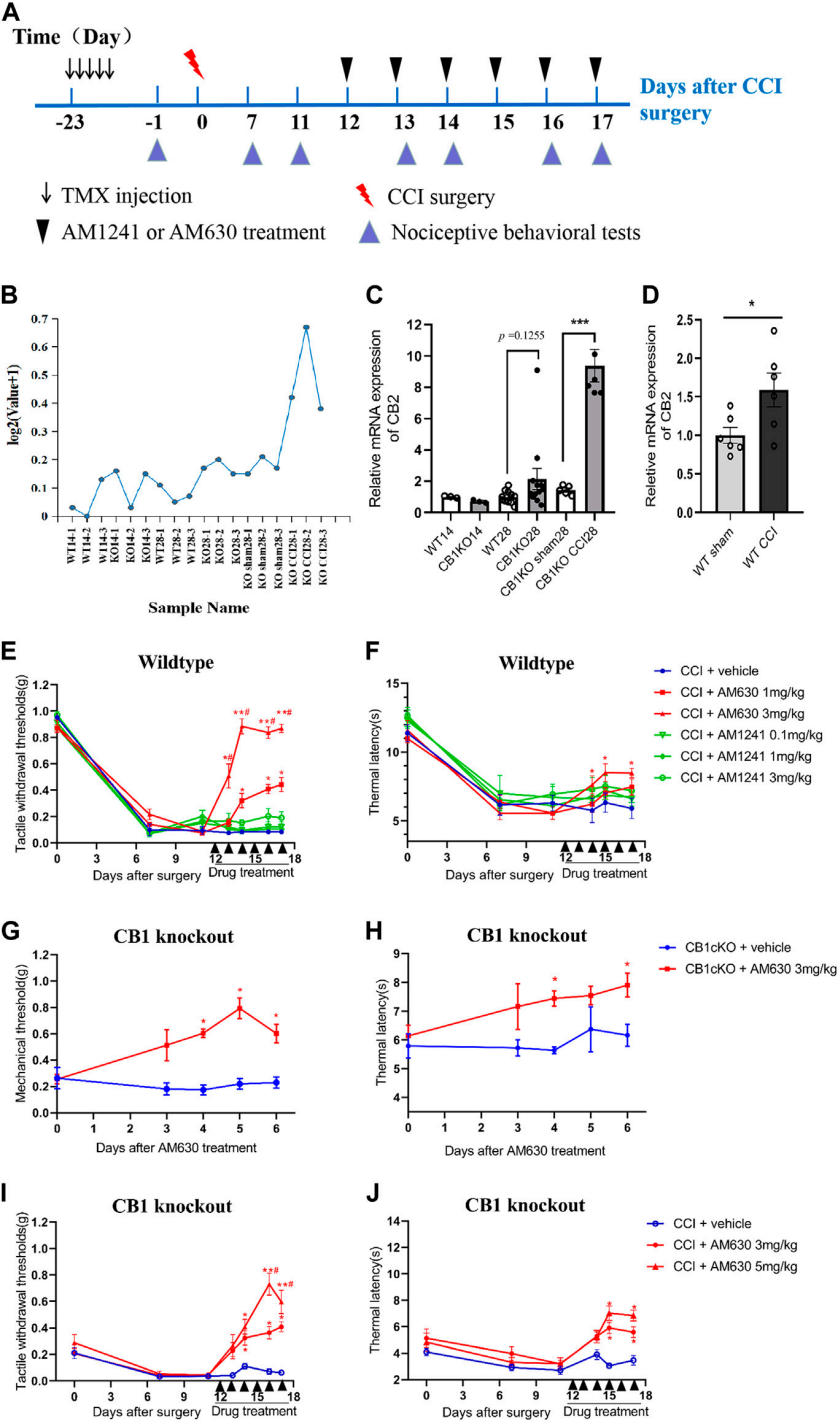
CB2 mediated neuropathic pain in CB1cKO mice subjected to chronic constriction injury (CCI). **(A)** Experimental flowchart. **(B)** RNA-seq shows a broken line graph of the expression levels of CB2 in the dorsal root ganglion (DRG) in different phases of CB1 knockout and after CCI surgery. **(C)** Real-time PCR data showing the mRNA level of CB2 in the DRG in different phases of CB1 knockout and after CCI surgery (*n* = 3–5 samples/group). Gapdh was used as an endogenous control, and the mean value in the WT14 group was set to 1. ***p < 0.001, one-way ANOVA, Fisher’s test. **(D)** Real-time PCR data showing the mRNA level of CB2 in the DRG of wild-type (WT) mice at 14 days after sham or CCI surgery (*n* = 6 each for CCI and 5 each for sham). Gapdh was used as an endogenous control gene, and the mean value of WT sham group was set to 1. *p < 0.05, two-tailed *t* test. **(E, F)** Time course of the tactile withdrawal thresholds **(E)** and thermal latency **(F)** in WT CCI mice treated with vehicle, AM630 (1, 3 mg/kg/day for 6 consecutive days), or AM1241 (0.1, 1, 3 mg/kg/day for 6 consecutive days) 11 days after CCI surgery (*n* = 6 each group). *p < 0.05, **p < 0.01, compared with WT CCI plus vehicle group; #p < 0.05, compared with WT CCI plus 1 mg/kg AM630 (two-way ANOVA with Bonferroni’s *post-hoc* test). **(G, H)** Time course of the tactile withdrawal thresholds **(G)** and thermal latency **(H)** in WT CCI and CB1cKO mice treated with vehicle, AM630 (3, 5 mg/kg/day for 6 consecutive days) 11 days after CCI surgery (*n* = 5 each group), *p < 0.05, **p < 0.01, compared with the respective CCI plus vehicle; #p < 0.05, compared with CB1cKO CCI plus 3 mg/kg AM630 (two-way ANOVA followed by Bonferroni’s *post-hoc* test). All data represent mean ± SEM.

From the sequencing results, we found that the expression of CB2 in DRG was 5.7 times higher in CB1cKO mice after CCI (Figure 8B, p < 0.001). qPCR results were consistent with unbiased RNA-Seq data (p < 0.001, Figure 8C). In contrast, in WT mice, there was only a slight increase (1.45 times) of CB2 in DRG after CCI modeling (Figure 8D, p < 0.05). It indicated that CB2 was a potential downstream molecule of CB1, and upregulation of expression of CB2 in DRG may play an important role in neuropathic pain after CB1 deletion.

We next determined whether intervention with CB2 may improve neuropathic pain. We treated WT CCI mice (12 days after CCI surgery) with daily intraperitoneal injection of AM630 (a highly specific CB2 antagonist) and AM1241 (a CB2 agonist) for 6 days. An experimental design time line with the day and time of all manipulations is presented in Figure 8A. In WT mice subjected to CCI, 1 and 3 mg/kg of AM630 significantly increased the MWT and thermal latency (Figures 8E, F; *p < 0.05, *n* = 5 mice). The analgesic effect was better when the dosage of AM630 was 3 mg/kg, and MWT can return to normal after the third day (Figure 8E, #p < 0.05, *n* = 5 mice). In contrast, 0.1, 1, and 3 mg/kg AM1241 had no significant effect on the tactile allodynia and thermal hyperalgesia (Figures 8E, F; p > 0.05, *n* = 5 mice). Thus, our results suggested that CB2 activation promotes the development of neuropathic pain; antagonizing CB2 can play an analgesic effect.

Although both RNA sequencing and qPCR results showed that DRG CB2 expression was not significantly increased compared with WT mice in the late phase of CB1 knockout (Figure 8C, p = 0.1255, *n* = 10–12), we measured the effect of AM630 in CB1cKO mice (28 days after TMX induction without CCI modeling). Behavioral results showed that 3 mg/kg AM630 (once a day for 6 days) significantly increased the MWT and thermal latency in CB1cKO mice (Figures 8G, H; *n* = 5, *p < 0.05). It indicated that antagonizing CB2 by AM630 plays a good analgesic effect in CB1cKO mice without CCI modeling.

Furthermore, we measured the effect of AM630 in CB1cKO mice 12 days after surgery with daily intraperitoneal injection for 6 days. Likewise, AM630 at both 3 and 5 mg/kg significantly increased the MWT and thermal latency in CB1cKO mice 2 weeks after CCI, and compared with 3 mg/kg of AM630, the dose of 5 mg/kg had better analgesic effect (Figures 8I, J; #p < 0.05, *n* = 5 mice). From these data, we can confirm that CB2 was a downstream gene of CB1, and CB2 mediated the development of neuropathic pain. These data provided new evidence that CB2 in DRG was indispensable for nerve injury–induced neuropathic pain; antagonizing CB2 can play an analgesic effect on neuropathic pain.

### AM630 Ameliorates Neuropathic Pain by Inhibiting Overactivation of Astrocytes and Neuroinflammation

**FIGURE 9:**
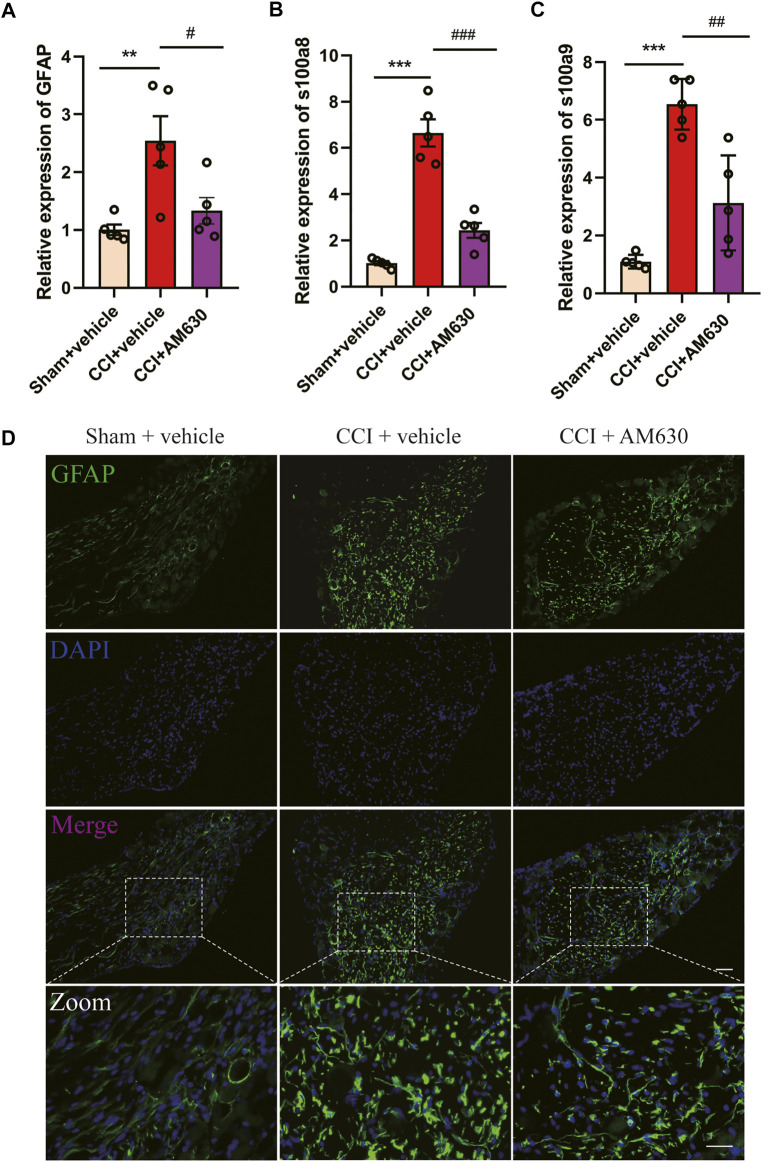
AM630 ameliorates neuropathic pain by inhibiting overactivation of astrocytes and neuroinflammation. **(A–C)** Real-time PCR data showing the mRNA level of glial fibrillary acidic protein (GFAP), s100a8, s100a9 (*n* = 5 samples/group); the endogenous reference control was gapdh, and the mean value of CB1cKO sham group was set to 1. *p < 0.05, **p < 0.01, ***p < 0.001, compared with CB1cKO sham + vehicle group; #p < 0.05, ##p < 0.01, ###p < 0.001, compared with CB1cKO chronic constriction injury (CCI) + vehicle group, one-way ANOVA with Bonferroni test. **(D)** Representative immunofluorescence images of GFAP (green) in dorsal root ganglion (DRG) display activation of astrocytes. ×20, scale bar, 100 μm; zoom, scale bar, 50 μm.

Since antagonizing CB2 by AM630 alleviated CCI-induced neuropathic pain, we wondered whether AM630 can inhibit glial overactivation or neuroinflammation in DRG induced by CCI. The mRNA expression of GFAP (an astrocyte marker), s100a8/s100a9 in DRG of CB1cKO CCI group was significantly higher than that of CB1cKO sham group (Figures 9A–C, *p < 0.01, *n* = 5). Compared with CB1cKO CCI group, AM630 significantly reduced the mRNA expression of GFAP, s100a8/s100a9 in DRG (Figures 9A–C, #p < 0.05, *n* = 5). Immunofluorescence staining of DRG also demonstrated that CCI significantly enlarged the cell body of astrocyte and upregulated GFAP expression in CB1cKO mice, which was significantly decreased by AM630 (Figure 9D). These results confirmed that in the condition of CB1 deletion in peripheral sensory neurons, after peripheral nerve injury, CB2 led to overactivation of glial cells and severe neuroinflammation, while antagonizing CB2 inhibited excessive activation of astrocytes and neuroinflammation, thus relieving neuropathic pain.

## Discussion

The development of neuropathic pain involves complicated processes, e.g., activation of certain pathways and aberrant gene expression. In this study, our data showed that CB1cKO mice presented persistent hyperalgesia and changed transcriptome expression in DRG in early and late phases of CB1cKO. CCI aggravated pain behavior and changed transcriptome expression (such as CB2) in DRG of CB1cKO mice. Finally, we found that interfering with downstream genes of CB1, such as antagonizing CB2, ameliorated neuropathic pain by inhibiting overactivation of astrocytes and neuroinflammation. Herein, our current data are the first to identify the transcriptome expression of CB1cKO in DRG before and after CCI modeling and provided new evidence that intervention with downstream genes of CB1 would be a potential treatment for neuropathic pain.

In neuropathic pain, cannabinoids produce analgesic effects primarily through activation of CB1 (Bridges et al., 2001). The mice that lacked CB1 in nociceptive (Nav1.8 promoter) sensory neurons presented tactile allodynia and thermal hyperalgesia (Agarwal et al., 2007). Consistently, in our study, CB1cKO mice also showed the above pain behavior in physiological conditions. Moreover, we found that CB1cKO mice after CCI modeling had exaggerated neuropathic pain compared to the WT CCI mice. In contrast, there was no significant difference between CB1cKO (in nociceptive sensory neurons) mice and WT littermates in pain behavior after spared nerve injury (SNI) (Agarwal et al., 2007), which probably results from a ceiling effect after SNI. Therefore, CCI had less damage to peripheral nerves and may be more sensitive to evaluate pain behavior. The present study showed that CB1 on peripheral sensory neurons mainly played a direct analgesic role.

Recent studies indicated that TMX not only activated Cre recombinase in transgenic mice but also resulted in various side effects and potentially confounding the phenotypic findings of the model itself (Carboneau et al., 2016; Falke et al., 2017; Hammad et al., 2018). In the present study, we also observed that TMX caused temporarily mechanical allodynia, thermal hyperalgesia, weight loss, and diarrhea in WT mice at the beginning of TMX induction. These symptoms gradually disappeared after the TMX withdrawal (from day-8 to day-14) except for thermal hyperalgesia. We speculated that these symptoms could be side effects induced by TMX, which disappeared as a result of the drug degradation, while thermal latency was more sensitive to TMX and may take longer to recover. Considering the experimental period, we suggested that a 2-week waiting period after TMX treatment may reduce confounding factors in subsequent experiments.

A great number of studies have investigated the functions of the endocannabinoid system in the regulation of metabolic homeostasis (He and Shi, 2017). Notably, in the early phase of CB1cKO, the functional pathways of DEGs are mainly enriched in material metabolism, energy balance, and complement and coagulation cascades. Besides these pathways, we also found the following genes changed, such as *Alb* (Vincenzetti et al., 2019), *Apoa1* (Bellei et al., 2017; Oehler et al., 2020a; Oehler et al., 2020b), *Nnat* (Chen et al., 2010), *Vgf* (Rizzi et al., 2008; Fairbanks et al., 2014), and *Sting1* (Donnelly et al., 2021), which may be associated with hyperalgesia in the early phase of CB1 knockdown. Interestingly, most DEGs involved in material metabolism and energy balance in the early phase of CB1cKO returned to normal after 2 weeks (the late phase of CB1cKO), including major urinary protein family and serine (or cysteine) peptidase inhibitor family. Since the above genes determine the survival of peripheral sensory neurons, they returned to normal after a short-term imbalance to maintain the basic physiological functions of DRG.

In the late phase of CB1 knockout, we observed a lot of DEGs, many of them have been reported in several sequencing articles of DRG of neuropathic pain models (Wu et al., 2016; Sun et al., 2020; Tang et al., 2020), including npy, atf3, Sprr1a, and Slc6a4. Furthermore, DEGs observed in the present profiling specifically targeted neuropeptide signaling (npy, npy1r, nts, Sprr1a, and Slc6a4), retrograde endocannabinoid signaling, ion transport, and pain regulation pathways. Due to the deletion of CB1 on DRG neurons, its inhibitory effect on presynaptic neurotransmitter release is weakened (Balezina et al., 2021), so the synthesis and release of presynaptic neurotransmitters increase, such as upregulation of npy, npy1r, and nts, which in turn causes the excitement of postsynaptic neurons and finally causes pain. Collectively, our results proved that the DEGs in the late phase of CB1 knockout correlated closely with neuropathic pain, which may provide new analgesic targets for neuropathic pain.

Neuroinflammation is a crucial mechanism in many neurological disorders. Injury to the peripheral sensory nerves leads to a neuroinflammatory response in the somatosensory pathway, especially from DRG to spinal cord, which results in neuropathic pain (Hu et al., 2020). Our results showed that CB1 was absent on peripheral sensory neurons; the immune response and neuroinflammation caused by peripheral nerve injury were more serious. Due to the lack of presynaptic inhibition of CB1 (Balezina et al., 2021; Patzke et al., 2021), peripheral nerve injury leads to glial overactivation and the release of a large amount of pain-causing substances and inflammatory mediators, which eventually causes exaggerated neuropathic pain, and the specific deep-level mechanism needs to be further studied.

Nerve injury profoundly reduced the mRNA level of CB1 at 5 days after spinal nerve ligation (SNL) but increased the mRNA expression of CB2 on 10 and 21 days but not 5 days after SNL in the rat DRG (Luo et al., 2020). Consistently, our study showed that the mRNA levels of CB2 of WT mice only slightly increased (1.45 times) on the 14th day after CCI, while the expression of CB2 of CB1cKO mice increased up to 6.5 times on the 14th day after CCI. It suggested that without the inhibitory effect of CB1, nerve injury can cause a large increase in the expression of CB2, which may contribute to the development of neuropathic pain.

Behavioral results suggested that AM630 has good analgesic effect on CB1cKO mice (28 days after TMX induction and no CCI modeling), which indicated that even if the expression of CB2 is not significantly increased after CB1 knockout, its function is activated, and antagonizing CB2 can relieve pain. Furthermore, AM630 also alleviated neuropathic pain induced by CCI in both WT mice and CB1KO mice. From this, we can confirm that CB2 is the downstream gene of CB1, and CB2 mediates the development of neuropathic pain. Since CB1 agonists had no analgesic effect on the maintenance of neuropathic pain (Luo et al., 2020), CB2 antagonists should be used to treat it without the central side effects of CB1 agonists. Besides CB2, other downstream genes of CB1 may also be used as an effective target for neuropathic pain.

CB2 is particularly attractive as a target due to the lack of the side effects such as psychotropic activity caused by CB1 agonists (Lutz, 2020). Activation of peripheral CB2 receptors is sufficient to produce antinociception to an acute thermal stimulus (Malan et al., 2001). Four days after L5 nerve transection, intrathecal administration of JWH015 (a CB2 agonist) has an analgesic effect (Landry et al., 2012). In contrast, our data suggested that in 12–17 days after CCI modeling, antagonizing the function of CB2 had a significant analgesic effect in both WT mice and CB1KO mice, while CB2 agonists leads to no analgesic effect. This discrepancy might result from different models and different administration times of CB2 agonists after nerve injury. Consistently, at the early stage before the onset of CNS injury-induced immunodeficiency syndrome (CIDS), CB2 activation has the potential to alleviate CNS injury by limiting neuroinflammation and preventing the development of CIDS, while at the later stage with already established CIDS, CB2 inhibition with AM630 also restored the peripheral leukocyte response to endotoxin to improve the patient’s outcome (Sultana et al., 2021). Our research found that in the maintenance phase of neuropathic pain, antagonizing CB2 can inhibit excessive activation of astrocytes and neuroinflammation that improves neuropathic pain induced by CCI.

In summary, our findings revealed that CB1 in peripheral sensory neurons mainly functioned as an endogenous analgesic factor. The action mechanisms of CB1cKO at early and late onsets of pain were different, as evidenced by our transcriptome sequencing results. CCI aggravated neuropathic pain and led to significant transcriptome profiling changes in DRG of CB1cKO mice. As a downstream target of CB1, CB2 worsened the neuropathic pain, and further use of CB2 antagonists reversed pain manifestations by inhibiting overactivation of astrocytes and neuroinflammation. These transcriptome profiling changes in DRG induced by CB1cKO and peripheral nerve injury could provide new directions and inspirations for developing novel drugs for neuropathic pain in the future.

## Data Availability

The data presented in the study are deposited in the NCBI repository, accession number PRJNA780970 (https://dataview.ncbi.nlm.nih.gov/object/PRJNA780970?reviewer=8b9hq4nt4j721tik2lbib4557u).

## Supplementary Material

Supplementary Figure S1 Agarose gel electrophoresis images of tail DNA of transgenic mice. CB1cKO mice are homozygous model of tissue/cell specific knockdown of CB1R, which required both LOXP element and advilin cre gene insertion. **(A)** To detect the presence of LOXP components in CB1-FLOX. The 344 bp band means the presence of LOXP elements. The mice are CB1-FLOX positive. The 244 bp band means that the loxp element is absent and means CB1-FLOX negative. This data showed that the transgenic mice numbered 822 to 837 are all CB1-FLOX positive. **(B)** To detect whether the advilin cre gene has been inserted. The 152 bp band indicates positive advilin Cre, and no band means advilin cre negative. Figure B shows that only 9 transgenic mice (numbered 825–827, 830–833, and 835–836) were advilin cre positive. **(C)** To test whether there is gene leakage in the mice’s tail (Whether CB1R is knocked out in the mice’s tail). If the transgenic mice had a gene leak, 350 bp bands (null) would appear, and no band means that there is no gene leak in the tail of the mouse. Figure C shows that no gene leakage occurred in the tail of transgenic mice numbered 822–837. With all these results mentioned above, there is a conclusion that 9 double-positive mice (numbered 825–827, 830–833, and 835–836) were successfully identified without gene leakage.
