# Supplementary material for: Transcriptomic Profiling in Mice With CB1 receptor Deletion in Primary Sensory Neurons Suggests New Analgesic Targets for Neuropathic Pain
Source: Front Pharmacol. 2022 Jan 3;12:781237. doi: 10.3389/fphar.2021.781237 (PMC8762320; doi:10.3389/fphar.2021.781237)
Supplement: Supplementary file 1 [file DataSheet1.docx]

Supplementary Material

# Supplementary Table 1

The PCR primers for identification of transgenic mice

| Gene name | Sequence |
| --- | --- |
| Cb1 flox-F1 | CCGTCATGTTAGCCATCTGCATTT |
| Cb1 flox-R1 | TCATGGCACGGCAGATAAGAACTT |
| Advillin cre-F | ACCCCGACTTTGTGATGTTTC |
| Advillin cre-R | CTGCCTGTCCCTGAACATG |
| Cb1-F1 | CCGTCATGTTAGCCATCTGCATTT |
| Cb1-R1 | GCACGGTGACAGTCACTATTTTA |

# Supplementary Table 2 PCR products for identification of transgenic mice

| PCR products | WT | Flox | null |
| --- | --- | --- | --- |
| Cb1 flox | 244bp | 341bp |  |
| Advillin cre | no bands | 152bp |  |
| Cb1 | 2438bp | 2600bp | 350bp |

# Supplementary Table 3 The primers for PCR

| Gene name | Sequence |
| --- | --- |
| cnr1-forward | CCTGCTGAACTCCACCGTGAAC |
| cnr1-reverse | CTGTGTTGTTGGCGTGCTTGTG |
| Cnr2-forward | CGTGATCTTCGCCTGCAACT |
| Cnr2-reverse | GTCAACAGCGGTTAGCAGCA |
| gfap-forward | GCCACGCTTCTCCTTGTCTC |
| gfap-reverse | CCATCATCTCTGCACGCTCG |
| s100a8-forward | TCACCATGCCCTCTACAAGAATGAC |
| s100a8-reverse | CCATCGCAAGGAACTCCTCGAAG |
| s100a9-forward | CCACCATCATCGACACCTTCCATC |
| s100a9-reverse | GCCAACTGTGCTTCCACCATTTG |

# Supplementary Table 4 The detailed information of the top 20 up-regulated and top 10 down-regulated mRNAs in the early phase of CB1R knockout(WT14 vs CB1cKO14).

| Up-regulated genes | | |  | Down-regulated genes | | |
| --- | --- | --- | --- | --- | --- | --- |
| Gene name/Gene Description | log2 | Information related to pain |  | Gene name/Gene Description | log2 | Information related to pain |
| Mup7（major urinary protein 7） | 8.35 |  |  | S100a9(S100 calcium binding protein A9 (calgranulin B)) | -1.32 | ***(Blom et al., 2020,** Forouzan et al., 2018) |
| Mup22（major urinary protein 22） | 7.46 |  |  | Ngp( neutrophilic granule protein) | -1.72 |  |
| Mup19（major urinary protein 19） | 7.45 |  |  | Cnr1( cannabinoid receptor 1) | -2.37 |  |
| Mup11（major urinary protein 11） | 6.73 |  |  | Mrpl35(mitochondrial ribosomal protein L35) | -0.52 |  |
| Mup14（major urinary protein14） | 6.70 |  |  | Fam234b(family with sequence similarity 234, member B) | -0.52 |  |
| Mup15（major urinary protein 15） | 5.97 |  |  | Nnat(neuronatin) | -0.52 | ***(Chen et al., 2010)** |
| Mup10（major urinary protein10） | 3.84 |  |  | Alkal2(ALK and LTK ligand 2) | -0.58 |  |
| Mup20（major urinary protein 20） | 2.74 |  |  | Vgf | -0.61 | ***(Fairbanks et al., 2014,** Rizzi et al., 2008) |
| Serpina1b（serine (or cysteine) preptidase inhibitor, clade A, member 1B） | 5.89 |  |  | Sting1(stimulator of interferon response cGAMP interactor 1) | -0.85 | ***(Donnelly et al., 2021,** Wang et al., 2021a) |
| Serpina1c（serine (or cysteine) peptidase inhibitor, clade A, member 1C） | 5.82 |  |  | Tmem267(transmembrane protein 267) | -0.78 |  |
| Serpina1a（serine (or cysteine) peptidase inhibitor, clade A, member 1a） | 5.41 |  |  |  |  |  |
| Serpina3k（serine (or cysteine) peptidase inhibitor, clade A, member 3k） | 4.01 |  |  |  |  |  |
| Serpina1d（serine (or cysteine) peptidase inhibitor, clade A, member 1d） | 6.14 |  |  |  |  |  |
| Pzp(alpha-2-macroglobulin like) | 5.80 | * |  |  |  |  |
| Alb(albumin) | 7.52 | ***(Vincenzetti et al., 2019)** |  |  |  |  |
| Ahsg( alpha-2-HS-glycoprotein) | 6.31 |  |  |  |  |  |
| Ces1d(carboxylesterase 1D) | 5.94 |  |  |  |  |  |
| Apoa1(apolipoprotein A-I) | 4.72 | ***(Bellei et al., 2017,** Oehler et al., 2020b, **Oehler et al., 2020a, Chen et al., 2020)** |  |  |  |  |
| Wdfy1( WD repeat and FYVE domain containing 1) | 1.56 |  |  |  |  |  |
| Tsfm(Ts translation elongation factor, mitochondrial) | 1.27 | #**(Traschütz et al., 2019)** |  |  |  |  |

*This gene is related to pain; #This gene is related to inflammation.

# Supplementary Table 5 The detailed information of the top 20 up-regulated and 20 down-regulated mRNAs in the late phase of CB1R knockdown (WT28 vs CB1cKO28).

| Up-regulated genes | | |  | Down-regulated genes | | |
| --- | --- | --- | --- | --- | --- | --- |
| Gene name/Gene Description | log2 | Information related to pain |  | Gene name/Gene Description | log2 | Information related to pain |
| Ucn( urocortin) | 5.98 |  |  | Slfn4( schlafen 4) | -3.52 |  |
| Sprr1a( small proline-rich protein 1A) | 4.70 | ***(Danaher et al., 2018)** |  | Ngp( neutrophilic granule protein) | -2.56 |  |
| Npy（neuropeptide Y） | 4.33 | ***(Nelson and Taylor, 2021)，** **(Alhadeff et al., 2018)，(Intondi et al., 2008)** |  | Ly6c2(lymphocyte antigen 6 complex, locus C2) | -2.33 |  |
| Gm9265( predicted gene 9265) | 1.66 |  |  | Ltf( lactotransferrin) | -2.30 |  |
| Gm42048( predicted gene 42048) | 1.08 |  |  | S100a8( S100 calcium binding protein A8 , calgranulin A) | -2.14 | ***(Forouzan et al., 2018)** |
| Slc6a4(solute carrier family 6 (neurotransmitter transporter, serotonin), member 4) | 2.77 | *(Lindstedt et al., 2011),**(Funke et al., 1990)** |  | Cnr1( cannabinoid receptor 1) | -2.11 | ***(Luo et al., 2020)** |
| Kcnk16( potassium channel, subfamily K, member 16) | 2.31 |  |  | Plvap(plasmalemma vesicle associated protein) | -2.10 |  |
| Atf3(activating transcription factor 3) | 2.15 | ***(Matsuura et al., 2013, Bourassa et al., 2020)** |  | S100a9( S100 calcium binding protein A9 (calgranulin B)) | -3.32 | *#**(Blom et al., 2020,** Forouzan et al., 2018) |
| Gpr151( G protein-coupled receptor 151) | 2.02 | *(Jiang et al., 2018) |  | Igf2( insulin-like growth factor 2) | -1.74 | ***(Grosman-Rimon et al., 2020)** |
| Gfap(glial fibrillary acidic protein) | 1.93 | ***(Guo et al., 2012, Hergenroeder et al., 2018)** |  | Ptgds( prostaglandin D2 synthase) | -1.60 | ***(Ouhaddi et al., 2020,** Xu et al., 2021) |
| Nts( neurotensin) | 1.78 | ***(Carr and Lipkowski, 1990, Feng et al., 2015)** |  | Chil3(chitinase-like 3) | 1.83 |  |
| Fos(FBJ osteosarcoma oncogene) | 1.40 | *****(Marvaldi et al., 2020,** Lv et al., 2019) |  | Cfd(complement factor D (adipsin)) | -1.55 |  |
| Tsfm(Ts translation elongation factor, mitochondrial) | 1.32 | #**(Traschütz et al., 2019)** |  | Ifitm1( interferon induced transmembrane protein 1) | -1.43 |  |
| Mettl1(methyltransferase like 1) | 1.26 |  |  | Fmo2( flavin containing monooxygenase 2) | -1.24 |  |
| Wdfy1( WD repeat and FYVE domain containing 1) | 1.26 |  |  | Elane( elastase, neutrophil expressed) | -1.14 |  |
| Slc7a11（solute carrier family 7 (cationic amino acid transporter, y+ system), member 11） | 1.22 | ***(Ungard et al., 2019, Miladinovic and Singh, 2019)** |  | Retnlg(resistin like gamma) | -1.07 | #**(Nagaev et al., 2006)** |
| Atp23(ATP23 metallopeptidase and ATP synthase assembly factor homolog) | 1.18 |  |  | Pmp2( peripheral myelin protein 2) | -1.06 |  |
| Nr4a3(nuclear receptor subfamily 4, group A, member 3) | 1.91 | ***(Zheng et al., 2017)** |  | Selp(P- selectin) | -1.01 | ***(Bai et al., 2014, Liu et al., 2007)** |
| Grk1( G protein-coupled receptor kinase 1) | 1.58 | ***(Li et al., 2019a)** |  | Ifi27l2a( interferon, alpha-inducible protein 27 like 2A) | 1.27 |  |
| Agtr1b(angiotensin II receptor, type 1b) |  | ***(Shepherd et al., 2018, Smith and Muralidharan, 2015)** |  | Camp | 1.18 | ***(Wang et al., 2021b)** |

*This gene is related to pain; #This gene is related to inflammation.

# Supplementary Table 6 The detailed information of the top 20 up-regulated and 20 down-regulated mRNAs in CB1cKO mice subjected to CCI (CB1cKO sham28 vs CB1cKO CCI28).

| Up-regulated genes | | |  | Down-regulated genes | | |
| --- | --- | --- | --- | --- | --- | --- |
| Gene name/Gene Description | log2 | Information related to pain |  | Gene name/Gene Description | log2 | Information related to pain |
| Cnr2(cannabinoid receptor 2) | 5.7 | ***(Lacombe et al., 1986, Luo et al., 2020)** |  | Calb2(calbindin 2) | -2.17 |  |
| Alb(albumin) | 8.61 | ***(Vincenzetti et al., 2019)** |  | Vstm2b(V-set and transmembrane domain containing 2B) | -1.16 | ***(Tang et al., 2020)** |
| Mmp8(Matrix metalloproteinases -8) | 9.05 | *#**(Cafferata et al., 2021, Tajerian and Clark, 2019)** |  | Kcng4(potassium voltage-gated channel, subfamily G, member 4) | -0.96 | ***(Lee et al., 2020)** |
| Ifitm6( interferon induced transmembrane protein 6) | 8.00 |  |  | Agtr1b(angiotensin II receptor, type 1b) | -0.79 | ***(Shepherd et al., 2018, Smith and Muralidharan, 2015)** |
| Ly6a2(lymphocyte antigen 6 complex, locus A2) | 7.63 |  |  | Tmem108(transmembrane protein 108) | -0.75 |  |
| Ngp(neutrophilic granule protein) | 7.44 |  |  | Mid1( midline 1) | -0.74 |  |
| S100a9( S100 calcium binding protein A9 (calgranulin B)) | 7.38 | *#**(Blom et al., 2020,** Forouzan et al., 2018) |  | Prrt3( proline-rich transmembrane protein 3) | -0.72 |  |
| S100a8( S100 calcium binding protein A8 (calgranulin A)) | 7.18 | ***(Forouzan et al., 2018)** |  | Omg( oligodendrocyte myelin glycoprotein) | -0.69 | *(Jarius et al., 2016, **Ishikawa et al., 2019)** |
| Ltf( lactotransferrin) | 7.11 |  |  | Gm765( predicted gene 765) | -0.68 |  |
| Slfn4( schlafen 4) | 6.93 |  |  | Fam234b(family with sequence similarity 234, member B) | -0.67 |  |
| Retnlg(resistin like gamma) | 6.77 | #(Nagaev et al., 2006) |  | Kcnq5( potassium voltage-gated channel, subfamily Q, member 5) | -0.64 | *(Manville and Abbott, 2018) |
| Ear6(eosinophil-associated, ribonuclease A family, member 6) | 6.71 |  |  | Kcnc3(potassium voltage gated channel, Shaw-related subfamily, member 3) | -0.63 |  |
| Chil3(chitinase-like 3) | 6.68 |  |  | Sema7a( sema domain, immunoglobulin domain (Ig), and GPI membrane anchor, (semaphorin) 7A) | -0.62 | #**(König et al., 2014,** Körner et al., 2021) |
| Reg3b( regenerating family member 3 beta) | 6.62 | #(Mei et al., 2019, Sarkar et al., 2020) |  | P2rx6(purinergic receptor P2X, ligand-gated ion channel, 6) | -0.61 | ***(Chen et al., 2016, Leng et al., 2019)** |
| Camp( cathelicidin antimicrobial peptide) | 6.61 | ***(Wang et al., 2021b)** |  | Rph3a(rabphilin 3A) | -0.60 |  |
| Pilrb1( paired immunoglobin-like type 2 receptor beta 1) | 6.50 |  |  | Thrsp( thyroid hormone responsive) | -0.60 |  |
| Ear1( eosinophil-associated, ribonuclease A family, member 1) | 6.46 |  |  | Patj( PATJ, crumbs cell polarity complex component) | -0.57 |  |
| Trem1( triggering receptor expressed on myeloid cells 1) | 6.35 | **#*(Li et al., 2019b,** Thankam et al., 2016, **Du et al., 2018)** |  | Kcnmb1(potassium large conductance calcium-activated channel, subfamily M, beta member 1) | -0.56 |  |
| Fcnb(ficolin B) | 6.27 |  |  | Faim2(Fas apoptotic inhibitory molecule 2) | -0.54 |  |
| Slpi( secretory leukocyte peptidase inhibitor) | 6.25 | ***(Shimoya et al., 2000)** |  | Pcdhac1( protocadherin alpha subfamily C, 1) | -0.54 |  |

*This gene is related to pain; #This gene is related to inflammation.

# Supplementary Table 7 Molecules enriched in top 9 KEGG pathway in the late phase of CB1R

# knockout

| KEGG pathway term | gene | Gene counts | P value |
| --- | --- | --- | --- |
| [04080](https://biosys.bgi.com/#/report/reanalysis/mrna/re-enrichment/gene/540f1e47b000/mus_musculus_10090.ncbi.gcf_000001635.26_grcm38.p6.v2005/kegg_pathway/false/_blank): Neuroactive ligand-receptor interaction | Npy, Gabra5, Agtr1a, Calca, Cckbr, Cnr1, Gabrg1, Gal, Gria4, Grid2, Grik1, Npy1r, P2ry1, Ucn, Lpar3, Nts | 16 | 0.02 |
| [04723](https://biosys.bgi.com/#/report/reanalysis/mrna/re-enrichment/gene/540f1e47b000/mus_musculus_10090.ncbi.gcf_000001635.26_grcm38.p6.v2005/kegg_pathway/false/_blank): Retrograde endocannabinoid signaling | Gabra5, Cnr1, Gabrg1, Gnb4, Gria4, Prkcb, Adcy2, Slc17a8, Mgll | 9 | 0.03 |
| [05032](https://biosys.bgi.com/#/report/reanalysis/mrna/re-enrichment/gene/540f1e47b000/mus_musculus_10090.ncbi.gcf_000001635.26_grcm38.p6.v2005/kegg_pathway/false/_blank): Morphine addiction | Grk2, Gabra5, Gabrg1, Gnb4, Prkcb, Adcy2, Pde11a | 7 | 0.03 |
| [04713](https://biosys.bgi.com/#/report/reanalysis/mrna/re-enrichment/gene/540f1e47b000/mus_musculus_10090.ncbi.gcf_000001635.26_grcm38.p6.v2005/kegg_pathway/false/_blank): Circadian entrainment | Gnb4, Gria4, Nos1, Prkcb, Adcy2, Cacna1i, Rps6ka5 | 7 | 0.03 |
| [05164](https://biosys.bgi.com/#/report/reanalysis/mrna/re-enrichment/gene/540f1e47b000/mus_musculus_10090.ncbi.gcf_000001635.26_grcm38.p6.v2005/kegg_pathway/false/_blank): Influenza A | Cdk4, Irf9, Prkcb, Stat2, Trim25, Tnfrsf1a, Oas1a, Irf7, Ifih1 | 9 | 0.04 |
| [04010](https://biosys.bgi.com/#/report/reanalysis/mrna/re-enrichment/gene/540f1e47b000/mus_musculus_10090.ncbi.gcf_000001635.26_grcm38.p6.v2005/kegg_pathway/false/_blank): MAPK signaling pathway | Bdnf, Csf1, Hspb1, Igf2, Jun, Ntrk1, Prkcb, Rasgrf2, Rasgrp1, Tnfrsf1a, Cacna1i, Rps6ka5 | 12 | 0.04 |
| [04724](https://biosys.bgi.com/#/report/reanalysis/mrna/re-enrichment/gene/540f1e47b000/mus_musculus_10090.ncbi.gcf_000001635.26_grcm38.p6.v2005/kegg_pathway/false/_blank): Glutamatergic synapse | Grk2, Gnb4, Gria4, Grik1, Prkcb, Adcy2, Slc17a8 | 7 | 0.04 |
| [04923](https://biosys.bgi.com/#/report/reanalysis/mrna/re-enrichment/gene/540f1e47b000/mus_musculus_10090.ncbi.gcf_000001635.26_grcm38.p6.v2005/kegg_pathway/false/_blank): Regulation of lipolysis in adipocytes | Npy, Npy1r, Adcy2, Mgll, Irs2 | 5 | 0.04 |
| [04970](https://biosys.bgi.com/#/report/reanalysis/mrna/re-enrichment/gene/540f1e47b000/mus_musculus_10090.ncbi.gcf_000001635.26_grcm38.p6.v2005/kegg_pathway/false/_blank): Salivary secretion | Atp1b3, Fxyd2, Nos1, Prkcb, Adcy2, Atp2b4 | 6 | 0.04 |

# Supplementary Table 8 Molecules enriched in top 8 biological process in the late phase of CB1R knockout

| Go-process term | gene | Gene counts | P value |
| --- | --- | --- | --- |
| GO:0007399-nervous system development | Bdnf, Chl1, Cntn1, Epha5, Gal, Gap43, Gfra2, Gfra3, Grik1, Ldb2, Myt1, Ntrk1, Sema6a, Sox11, Unc119, Scn3b, Plxna4, Tnik, Pcdh18, Mdga1' | 20 | 1.44E-06 |
| GO:0007411-axon guidance | Bdnf, Chl1, Epha5, Gap43, Gfra3, Ntrk1, Pou4f2, Sema6a, Wnt5a, Plxna4, Nrcam, Flrt3 | 12 | 3.75E-06 |
| GO:0007631-feeding behavior | Npy, Bdnf, Calca, Gal, Npy1r, Ucn | 6 | 3.14E-06 |
| GO:0007218-neuropeptide signaling pathway | Npy, Calca, Ecel1, Gal, Npy1r, Ucn, Gpr149, Cartpt, Nts | 9 | 9.18E-06 |
| GO:0007605-sensory perception of sound | Aqp1, Cnr1, Ndn, Npy1r, Ntrk1, P2ry1, Prx, Scn3b | 8 | 6.85E-06 |
| GO:0019233-sensory perception of pain | Cckbr, Nos1, Mgll, Tafa4, Acpp, Tmem100 | 6 | 7.54E-06 |
| GO:0042552-myelination | Mal2, Mal, Mbp, Pmp22, Ugt8a, Pllp, Tnfrsf21 | 7 | 7.17E-06 |
| [GO:0045087](http://amigo.geneontology.org/amigo/term/GO:0045087#/report/reanalysis/mrna/re-enrichment/gene/540f44c9c000/mus_musculus_10090.ncbi.gcf_000001635.26_grcm38.p6.v2005/go_p/false/_blank)-innate immune response | Npy, C4b, Cd24a, Csf1, Ifit2, Lgals3, Ltf, S100a8, S100a9, Tifa, Trim25, Oas1a, Irf7, Wfdc2, Ifih1, Zc3hav1,Dhx58 | 17 | 1.67E-05 |

**Supplementary Table 9 Molecules enriched in the top 7 KEGG pathway in CB1cKO mice subjected to CCI**

| KEGG pathway term | gene | Gene counts | P value |
| --- | --- | --- | --- |
| 5150: Staphylococcus aureus infection | Cfd, C3, C3ar1, C4b, Camp, Fcgr1, Fcgr2b, Fcgr3, Fpr2, Fpr1, H2-Ab1, Cfb, H2-Eb1, Icam1, Itgal, Itgam, Itgb2, Itgb2l, Krt19, Selplg | 20 | 5.23E-12 |
| 5140: Leishmaniasis | C3, Cyba, Cybb, Fcgr1, Fcgr3, H2-Ab1, H2-Eb1, Ptpn6, Itgam, Itgb2, Itgb2l, Ncf2, Tlr2 | 13 | 1.14E-08 |
| 4145: Phagosome | C3, Ctss, Cyba, Cybb, Fcgr1, Fcgr2b, Fcgr3, H2-Ab1, H2-Eb1, Itgam, Itgb2, Itgb2l, Mrc1, Ncf2, Nos1, Tuba1c, Tlr2, Tubb6, Tubb2b | 19 | 8.35E-08 |
| 5152: Tuberculosis | C3, Casp3, Camp, Ctss, Fcgr1, Fcgr2b, Fcgr3, H2-Ab1, H2-Eb1, Itgam, Itgb2, Itgb2l, Lbp, Lsp1, Mrc1, Syk, Tlr1, Tlr2, Clec4e | 19 | 8.35E-08 |
| 4380: Osteoclast differentiation | Acp5, Csf1, Csf1r, Cyba, Fcgr1, Fcgr2b, Fcgr3, Lilrb4a, Il1r1, Ncf2, Pirb, Spi1, Syk, Trem2 |  | 3.17E-08 |
| 4662: B cell receptor signaling pathway | Cd72, Fcgr2b, Lilrb4a, Ptpn6, Inpp5d, Lyn, Pirb, Rac2, Syk, Vav1,Pik3ap1 |  | 1.9E-08 |
| 4666: Fc gamma R-mediated phagocytosis | Fcgr1, Fcgr2b, Hck, Inpp5d, Lyn, Ptprc, Rac2, Syk, Vav1, Was, Dock2' |  | 8.9E-07 |

**Supplementary Table 10 Molecules enriched in the top 7 biological process in CB1cKO mice subjected to CCI**

| Go-process Term | gene | Gene counts | P value |
| --- | --- | --- | --- |
| GO:0045087-innate immune response | Wfdc17, Pld4, Npy, Cfd, C3, C4b, Camp, Csf1, Csf1r, Cyba, Cybb, Fcgr1, Fcnb, Cfb, Hck, Arhgef2, Lbp, Lcn2, Lgals3 | 52 | 3.91E-25 |
| GO:0002376-immune system process | Pld4, Cfd, C3, Csf1, Csf1r, Cd300c2, Fcgr1, Fcnb, Lilrb4a, H2-Ab1, Cfb, H2-Eb1, Hck, Hp, Irf8, Inpp5d, Arhgef2, Lbp, Lcn2 | 53 | 1.07E-23 |
| GO:0006954-inflammatory response | Pld4, Adam8, Agtr1b, C3, C3ar1, C4b, Chil3, Ccr1, Ccr2, Crh, Csf1, Csf1r, Cyba, Cybb, Fpr2, Fpr1, Gal, Lilrb4a, Hck | 44 | 1.65E-21 |
| GO:0009617-response to bacterium | Mnda, Ly6a, Cfd, Fabp4, C3, Car3, Cyp2e1, Fcgr1, Fcgr2b, Hp, Irf8, Ifi203, Ifi204, Lcn2, Myo1f, Reg3b, Trim30a, Slfn2, Slfn4 | 29 | 1.75E-17 |
| GO:0030593-neutrophil chemotaxis | Nckap1, Csf3r, Fcgr3, Itgam, Itgb2, Lgals3, S100a8, S100a9, Ccl12, Ccl6, Ccl7, Ccl8, Ccl9, Spp1, Syk, Vav1, Trem1 | 17 | 3.00E-13 |
| GO:0006935-chemotaxis | Nckap1l, C3ar1, Ccr1, Ccr2, Ear2, Fpr2, Fpr1, Lsp1, Rac2, S100a8, S100a9, Ccl12, Ccl6, Ccl7, Ccl8, Ccl9, Pik3cg, Dock2, Hmgb2 | 19 | 5.12E-11 |
| GO:0042742-defense response to bacterium | Chga, Camp, Epx, Fcgr1, Fpr2, Hp, Irf8, Lbp, Lcn2, Lyz2, Mpeg1, Prg2, Stab1, S100a11, Slpi, Spn, Syk, Pglyrp1, Plac8 | 21 | 1.01E-10 |

**References:**

Alhadeff, A.L., Su, Z., Hernandez, E., Klima, M.L., Phillips, S.Z., Holland, R.A., Guo, C., Hantman, A.W., De Jonghe, B.C., and Betley, J.N. (2018). A Neural Circuit for the Suppression of Pain by a Competing Need State. *Cell* 173**,** 140-152.e115.

Bai, Y.M., Chiou, W.F., Su, T.P., Li, C.T., and Chen, M.H. (2014). Pro-inflammatory cytokine associated with somatic and pain symptoms in depression. *J Affect Disord* 155**,** 28-34.

Bellei, E., Vilella, A., Monari, E., Bergamini, S., Tomasi, A., Cuoghi, A., Guerzoni, S., Manca, L., Zoli, M., and Pini, L.A. (2017). Serum protein changes in a rat model of chronic pain show a correlation between animal and humans. *Sci Rep* 7**,** 41723.

Blom, A.B., Van Den Bosch, M.H., Blaney Davidson, E.N., Roth, J., Vogl, T., Van De Loo, F.A., Koenders, M., Van Der Kraan, P.M., Geven, E.J., and Van Lent, P.L. (2020). The alarmins S100A8 and S100A9 mediate acute pain in experimental synovitis. *Arthritis Res Ther* 22**,** 199.

Bourassa, V., Deamond, H., Yousefpour, N., Fitzcharles, M.A., and Ribeiro-Da-Silva, A. (2020). Pain-related behavior is associated with increased joint innervation, ipsilateral dorsal horn gliosis, and dorsal root ganglia activating transcription factor 3 expression in a rat ankle joint model of osteoarthritis. *Pain Rep* 5**,** e846.

Cafferata, E.A., Monasterio, G., Castillo, F., Carvajal, P., Flores, G., Díaz, W., Fuentes, A.D., and Vernal, R. (2021). Overexpression of MMPs, cytokines, and RANKL/OPG in temporomandibular joint osteoarthritis and their association with joint pain, mouth opening, and bone degeneration: A preliminary report. *Oral Dis* 27**,** 970-980.

Carr, D.B., and Lipkowski, A.W. (1990). Neuropeptides and pain. *Agressologie* 31**,** 173-177.

Chen, C.P.C., Hsu, C.C., Huang, S.C., Lin, M.Y., Chen, J.L., and Lin, S.Y. (2020). The application of thermal oscillation method to augment the effectiveness of autologous platelet rich plasma in treating elderly patients with knee osteoarthritis. *Exp Gerontol* 142**,** 111120.

Chen, K.H., Yang, C.H., Cheng, J.T., Wu, C.H., Sy, W.D., and Lin, C.R. (2010). Altered neuronatin expression in the rat dorsal root ganglion after sciatic nerve transection. *J Biomed Sci* 17**,** 41.

Chen, L., Liu, Y.W., Yue, K., Ru, Q., Xiong, Q., Ma, B.M., Tian, X., and Li, C.Y. (2016). Differential expression of ATP-gated P2X receptors in DRG between chronic neuropathic pain and visceralgia rat models. *Purinergic Signal* 12**,** 79-87.

Danaher, R.J., Zhang, L., Donley, C.J., Laungani, N.A., Hui, S.E., Miller, C.S., and Westlund, K.N. (2018). Histone deacetylase inhibitors prevent persistent hypersensitivity in an orofacial neuropathic pain model. *Mol Pain* 14**,** 1744806918796763.

Donnelly, C.R., Jiang, C., Andriessen, A.S., Wang, K., Wang, Z., Ding, H., Zhao, J., Luo, X., Lee, M.S., Lei, Y.L., Maixner, W., Ko, M.C., and Ji, R.R. (2021). STING controls nociception via type I interferon signalling in sensory neurons. *Nature* 591**,** 275-280.

Du, C., Peng, L., Kou, G., Wang, P., Lu, L., and Li, Y. (2018). Assessment of Serum sTREM-1 as a Marker of Subclinical Inflammation in Diarrhea-Predominant Patients with Irritable Bowel Syndrome. *Dig Dis Sci* 63**,** 1182-1191.

Fairbanks, C.A., Peterson, C.D., Speltz, R.H., Riedl, M.S., Kitto, K.F., Dykstra, J.A., Braun, P.D., Sadahiro, M., Salton, S.R., and Vulchanova, L. (2014). The VGF-derived peptide TLQP-21 contributes to inflammatory and nerve injury-induced hypersensitivity. *Pain* 155**,** 1229-1237.

Feng, Y.P., Wang, J., Dong, Y.L., Wang, Y.Y., and Li, Y.Q. (2015). The roles of neurotensin and its analogues in pain. *Curr Pharm Des* 21**,** 840-848.

Forouzan, A., Masoumi, K., Rahim, F., Moezzi, M., Khavanin, A., Ranjbari, N., Amal Saki, M., Fallah Amoli, A., Akhiani, N., and Ghourchian, F. (2018). Diagnostic Accuracy of Serum and Urine S100A8/A9 and Serum Amyloid A in Probable Acute Abdominal Pain at Emergency Department. *Dis Markers* 2018**,** 6457347.

Funke, H.J., Moritz, E., Hellstern, K., and Malanowski, H. (1990). Moclobemide versus clomipramine in the treatment of depression: a single-centre study, Federal Republic of Germany. *Acta Psychiatr Scand Suppl* 360**,** 46-47.

Grosman-Rimon, L., Vadasz, B., Parkinson, W., Clarke, H., Katz, J.D., and Kumbhare, D. (2020). The Levels of Insulin-Like Growth Factor in Patients with Myofascial Pain Syndrome and in Healthy Controls. *Pm r*.

Guo, J., Jia, D., Jin, B., Xu, F., Yuan, X., and Shen, H. (2012). Effects of glial cell line-derived neurotrophic factor intrathecal injection on spinal dorsal horn glial fibrillary acidic protein expression in a rat model of neuropathic pain. *Int J Neurosci* 122**,** 388-394.

Hergenroeder, G.W., Redell, J.B., Choi, H.A., Schmitt, L., Donovan, W., Francisco, G.E., Schmitt, K., Moore, A.N., and Dash, P.K. (2018). Increased Levels of Circulating Glial Fibrillary Acidic Protein and Collapsin Response Mediator Protein-2 Autoantibodies in the Acute Stage of Spinal Cord Injury Predict the Subsequent Development of Neuropathic Pain. *J Neurotrauma* 35**,** 2530-2539.

Intondi, A.B., Dahlgren, M.N., Eilers, M.A., and Taylor, B.K. (2008). Intrathecal neuropeptide Y reduces behavioral and molecular markers of inflammatory or neuropathic pain. *Pain* 137**,** 352-365.

Ishikawa, H., Kezuka, T., Shikishima, K., Yamagami, A., Hiraoka, M., Chuman, H., Nakamura, M., Hoshi, K., Goseki, T., Mashimo, K., Mimura, O., Yoshitomi, T., and Tanaka, K. (2019). Epidemiologic and Clinical Characteristics of Optic Neuritis in Japan. *Ophthalmology* 126**,** 1385-1398.

Jarius, S., Ruprecht, K., Kleiter, I., Borisow, N., Asgari, N., Pitarokoili, K., Pache, F., Stich, O., Beume, L.A., Hümmert, M.W., Ringelstein, M., Trebst, C., Winkelmann, A., Schwarz, A., Buttmann, M., Zimmermann, H., Kuchling, J., Franciotta, D., Capobianco, M., Siebert, E., Lukas, C., Korporal-Kuhnke, M., Haas, J., Fechner, K., Brandt, A.U., Schanda, K., Aktas, O., Paul, F., Reindl, M., and Wildemann, B. (2016). MOG-IgG in NMO and related disorders: a multicenter study of 50 patients. Part 2: Epidemiology, clinical presentation, radiological and laboratory features, treatment responses, and long-term outcome. *J Neuroinflammation* 13**,** 280.

Jiang, B.C., Zhang, W.W., Yang, T., Guo, C.Y., Cao, D.L., Zhang, Z.J., and Gao, Y.J. (2018). Demethylation of G-Protein-Coupled Receptor 151 Promoter Facilitates the Binding of Krüppel-Like Factor 5 and Enhances Neuropathic Pain after Nerve Injury in Mice. *J Neurosci* 38**,** 10535-10551.

König, K., Marth, L., Roissant, J., Granja, T., Jennewein, C., Devanathan, V., Schneider, M., Köhler, D., Zarbock, A., and Rosenberger, P. (2014). The plexin C1 receptor promotes acute inflammation. *Eur J Immunol* 44**,** 2648-2658.

Körner, A., Bernard, A., Fitzgerald, J.C., Alarcon-Barrera, J.C., Kostidis, S., Kaussen, T., Giera, M., and Mirakaj, V. (2021). Sema7A is crucial for resolution of severe inflammation. *Proc Natl Acad Sci U S A* 118.

Lacombe, C., Corraze, G., and Nibbelink, M. (1986). The effect of cholestyramine on apolipoproteins in cholesterol-fed rabbits. *Artery* 13**,** 203-212.

Lee, M.C., Nahorski, M.S., Hockley, J.R.F., Lu, V.B., Ison, G., Pattison, L.A., Callejo, G., Stouffer, K., Fletcher, E., Brown, C., Drissi, I., Wheeler, D., Ernfors, P., Menon, D., Reimann, F., Smith, E.S.J., and Woods, C.G. (2020). Human Labor Pain Is Influenced by the Voltage-Gated Potassium Channel K(V)6.4 Subunit. *Cell Rep* 32**,** 107941.

Leng, C., Chen, L., and Li, C. (2019). Alteration of P2X1-6 receptor expression in retrograde Fluorogold-labeled DRG neurons from rat chronic neuropathic pain model. *Biomed Rep* 10**,** 225-230.

Li, H., Wan, H.Q., Zhao, H.J., Luan, S.X., and Zhang, C.G. (2019a). Identification of candidate genes and miRNAs associated with neuropathic pain induced by spared nerve injury. *Int J Mol Med* 44**,** 1205-1218.

Li, Z., Wu, F., Xu, D., Zhi, Z., and Xu, G. (2019b). Inhibition of TREM1 reduces inflammation and oxidative stress after spinal cord injury (SCI) associated with HO-1 expressions. *Biomed Pharmacother* 109**,** 2014-2021.

Lindstedt, F., Lonsdorf, T.B., Schalling, M., Kosek, E., and Ingvar, M. (2011). Perception of thermal pain and the thermal grill illusion is associated with polymorphisms in the serotonin transporter gene. *PLoS One* 6**,** e17752.

Liu, Y.F., Yu, H.M., Zhang, C., Yang, R.X., Yan, F.F., Liu, Y., Zhang, Y., and Zhao, Y.X. (2007). Effects of Quyu Xiaoban capsules on clinical outcomes and platelet activation and aggregation in patients with unstable angina pectoris. *J Altern Complement Med* 13**,** 571-576.

Luo, Y., Zhang, J., Chen, L., Chen, S.R., Chen, H., Zhang, G., and Pan, H.L. (2020). Histone methyltransferase G9a diminishes expression of cannabinoid CB(1) receptors in primary sensory neurons in neuropathic pain. *J Biol Chem* 295**,** 3553-3562.

Lv, S.Y., Cui, B., Yang, Y., Du, H., Zhang, X., Zhou, Y., Ye, W., Nie, X., Li, Y., Wang, Q., Chen, W.D., and Wang, Y.D. (2019). Spexin/NPQ Induces FBJ Osteosarcoma Oncogene (Fos) and Produces Antinociceptive Effect against Inflammatory Pain in the Mouse Model. *Am J Pathol* 189**,** 886-899.

Manville, R.W., and Abbott, G.W. (2018). Gabapentin Is a Potent Activator of KCNQ3 and KCNQ5 Potassium Channels. *Mol Pharmacol* 94**,** 1155-1163.

Marvaldi, L., Panayotis, N., Alber, S., Dagan, S.Y., Okladnikov, N., Koppel, I., Di Pizio, A., Song, D.A., Tzur, Y., Terenzio, M., Rishal, I., Gordon, D., Rother, F., Hartmann, E., Bader, M., and Fainzilber, M. (2020). Importin α3 regulates chronic pain pathways in peripheral sensory neurons. *Science* 369**,** 842-846.

Matsuura, Y., Ohtori, S., Iwakura, N., Suzuki, T., Kuniyoshi, K., and Takahashi, K. (2013). Expression of activating transcription factor 3 (ATF3) in uninjured dorsal root ganglion neurons in a lower trunk avulsion pain model in rats. *Eur Spine J* 22**,** 1794-1799.

Mei, Y., Fang, C., Ding, S., Liu, X., Hu, J., Xu, J., and Mei, Q. (2019). PAP-1 ameliorates DSS-induced colitis with involvement of NLRP3 inflammasome pathway. *Int Immunopharmacol* 75**,** 105776.

Miladinovic, T., and Singh, G. (2019). Spinal microglia contribute to cancer-induced pain through system x(C) (-)-mediated glutamate release. *Pain Rep* 4**,** e738.

Nagaev, I., Bokarewa, M., Tarkowski, A., and Smith, U. (2006). Human resistin is a systemic immune-derived proinflammatory cytokine targeting both leukocytes and adipocytes. *PLoS One* 1**,** e31.

Nelson, T.S., and Taylor, B.K. (2021). Targeting spinal neuropeptide Y1 receptor-expressing interneurons to alleviate chronic pain and itch. *Prog Neurobiol* 196**,** 101894.

Oehler, B., Brack, A., Blum, R., and Rittner, H.L. (2020a). Pain Control by Targeting Oxidized Phospholipids: Functions, Mechanisms, Perspectives. *Front Endocrinol (Lausanne)* 11**,** 613868.

Oehler, B., Kloka, J., Mohammadi, M., Ben-Kraiem, A., and Rittner, H.L. (2020b). D-4F, an ApoA-I mimetic peptide ameliorating TRPA1-mediated nocifensive behaviour in a model of neurogenic inflammation. *Mol Pain* 16**,** 1744806920903848.

Ouhaddi, Y., Najar, M., Paré, F., Lussier, B., Urade, Y., Benderdour, M., Pelletier, J.P., Martel-Pelletier, J., and Fahmi, H. (2020). L-PGDS deficiency accelerated the development of naturally occurring age-related osteoarthritis. *Aging (Albany NY)* 12**,** 24778-24797.

Rizzi, R., Bartolomucci, A., Moles, A., D'amato, F., Sacerdote, P., Levi, A., La Corte, G., Ciotti, M.T., Possenti, R., and Pavone, F. (2008). The VGF-derived peptide TLQP-21: a new modulatory peptide for inflammatory pain. *Neurosci Lett* 441**,** 129-133.

Sarkar, S., Nguyen, H.M., Malovic, E., Luo, J., Langley, M., Palanisamy, B.N., Singh, N., Manne, S., Neal, M., Gabrielle, M., Abdalla, A., Anantharam, P., Rokad, D., Panicker, N., Singh, V., Ay, M., Charli, A., Harischandra, D., Jin, L.W., Jin, H., Rangaraju, S., Anantharam, V., Wulff, H., and Kanthasamy, A.G. (2020). Kv1.3 modulates neuroinflammation and neurodegeneration in Parkinson's disease. *J Clin Invest* 130**,** 4195-4212.

Shepherd, A.J., Mickle, A.D., Golden, J.P., Mack, M.R., Halabi, C.M., De Kloet, A.D., Samineni, V.K., Kim, B.S., Krause, E.G., Gereau, R.W.T., and Mohapatra, D.P. (2018). Macrophage angiotensin II type 2 receptor triggers neuropathic pain. *Proc Natl Acad Sci U S A* 115**,** E8057-e8066.

Shimoya, K., Moriyama, A., Ogata, I., Nobunaga, T., Koyama, M., Azuma, C., and Murata, Y. (2000). Increased concentrations of secretory leukocyte protease inhibitor in peritoneal fluid of women with endometriosis. *Mol Hum Reprod* 6**,** 829-834.

Smith, M.T., and Muralidharan, A. (2015). Targeting angiotensin II type 2 receptor pathways to treat neuropathic pain and inflammatory pain. *Expert Opin Ther Targets* 19**,** 25-35.

Tajerian, M., and Clark, J.D. (2019). Spinal matrix metalloproteinase 8 regulates pain after peripheral trauma. *J Pain Res* 12**,** 1133-1138.

Tang, S., Jing, H., Huang, Z., Huang, T., Lin, S., Liao, M., and Zhou, J. (2020). Identification of key candidate genes in neuropathic pain by integrated bioinformatic analysis. *J Cell Biochem* 121**,** 1635-1648.

Thankam, F.G., Dilisio, M.F., Dietz, N.E., and Agrawal, D.K. (2016). TREM-1, HMGB1 and RAGE in the Shoulder Tendon: Dual Mechanisms for Inflammation Based on the Coincidence of Glenohumeral Arthritis. *PLoS One* 11**,** e0165492.

Traschütz, A., Hayer, S.N., Bender, B., Schöls, L., Biskup, S., and Synofzik, M. (2019). TSFM mutations cause a complex hyperkinetic movement disorder with strong relief by cannabinoids. *Parkinsonism Relat Disord* 60**,** 176-178.

Ungard, R.G., Linher-Melville, K., Nashed, M.G., Sharma, M., Wen, J., and Singh, G. (2019). xCT knockdown in human breast cancer cells delays onset of cancer-induced bone pain. *Mol Pain* 15**,** 1744806918822185.

Vincenzetti, S., Pucciarelli, S., Huang, Y., Ricciutelli, M., Lambertucci, C., Volpini, R., Scuppa, G., Soverchia, L., Ubaldi, M., and Polzonetti, V. (2019). Biomarkers mapping of neuropathic pain in a nerve chronic constriction injury mice model. *Biochimie* 158**,** 172-179.

Wang, K., Donnelly, C.R., Jiang, C., Liao, Y., Luo, X., Tao, X., Bang, S., Mcginnis, A., Lee, M., Hilton, M.J., and Ji, R.R. (2021a). STING suppresses bone cancer pain via immune and neuronal modulation. *Nat Commun* 12**,** 4558.

Wang, Y., Dai, G., Jiang, L., Liao, S., and Xia, J. (2021b). Microarray analysis reveals an inflammatory transcriptomic signature in peripheral blood for sciatica. *BMC Neurol* 21**,** 50.

Xu, W., Ding, W., Sheng, H., Lu, D., Xu, X., and Xu, B. (2021). Dexamethasone Suppresses Radicular Pain Through Targeting the L-PGDS/PI3K/Akt Pathway in Rats With Lumbar Disc Herniation. *Pain Pract* 21**,** 64-74.

Zheng, X., Cong, J., Zhang, H., and Chu, X. (2017). Personalized analysis of pathway aberrance induced by sevoflurane and propofol. *Mol Med Rep* 16**,** 5312-5320.
